# NFAT1 Signaling Contributes to Bone Cancer Pain by Regulating IL‐18 Expression in Spinal Microglia

**DOI:** 10.1111/cns.70222

**Published:** 2025-02-17

**Authors:** Xuetai Chen, Ying Zeng, Zizhu Wang, Jixiang Zhu, Fengyun Liu, Mingxuan Zhu, Jiayi Zheng, Qingdaiyao Chen, Dongxu Zhai, Yangyang Chen, Jiayao Niu, Zhouya Xue, Guan Sun, Feng Li, Zhiqiang Pan

**Affiliations:** ^1^ Jiangsu Province Key Laboratory of Anesthesiology, Jiangsu Province Key Laboratory of Anesthesia and Analgesia Application Technology, NMPA Key Laboratory for Research and Evaluation of Narcotic and Psychotropic Drugs, Department of Anesthesiology The Yancheng Clinical College of Xuzhou Medical University, The First people’s Hospital of Yancheng Yancheng China; ^2^ Department of Anesthesiology Obstetrics and Gynecology Hospital of Fudan University Shanghai China; ^3^ Department of Neurosurgery The Yancheng Clinical College of Xuzhou Medical University, The First people's Hospital of Yancheng Yancheng Jiangsu China

**Keywords:** bone cancer pain, interleukin‐18, microglia, nuclear factor of activated T cells 1, p38 MAPK

## Abstract

**Aims:**

This study aimed to test the hypothesis that nuclear factor of activated T cells 1 (NFAT1) signaling contributes to bone cancer pain by regulating interleukin (IL)‐18 expression in spinal microglia.

**Methods:**

This study was performed on male mice using a Lewis lung carcinoma‐induced bone cancer pain model. Nociceptive behaviors were evaluated by measuring mechanical allodynia, thermal hyperalgesia, and spontaneous pain. Expression levels were measured via real‐time quantitative polymerase chain reaction, western blotting, and immunofluorescence analysis. The effect of pharmacologic intervention of spinal NFAT1/IL‐18 signaling on bone cancer pain was the primary outcome.

**Results:**

NFAT1 expression was upregulated in the spinal microglia after tumor inoculation. Pharmacological inhibition of NFAT1 upregulation prevented and reversed bone cancer‐related pain behaviors. In spinal microglia, NFAT1 inhibition decreased p38 MAPK phosphorylation and IL‐18 production. Blocking NFAT1 signaling suppressed tumor‐induced neuronal sensitization and microglial activation as well as activation of the N‐methyl‐D‐aspartate receptor and the subsequent Ca^2+^‐dependent signaling.

**Conclusion:**

Microglia NFAT1‐p38 signaling contributes to bone cancer pain through IL‐18‐mediated central sensitization in spinal microglia. NFAT1 could be a potential target for therapeutic intervention to prevent bone cancer pain.

## Graphical abstract

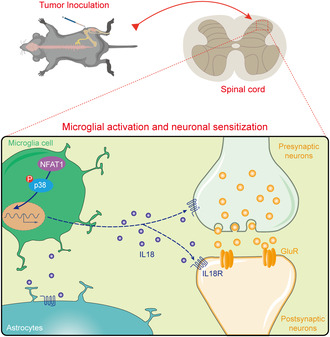

## 1 Introduction

Bone cancer pain is a disabling, chronic, and pathological state that seriously hampers physiological function and quality of life and involves complex pathophysiological mechanisms [1, 2, 3]. Most patients with cancer experience moderate‐to‐severe pain, which increases with disease progression, and 75%–90% of the patients with metastatic or advanced bone cancer experience severe cancer pain [4, 5]. Traditional analgesics, such as opioids, have many limitations, including the potential for addiction, drug resistance, and adverse effects such as dizziness and vomiting [6]. Therefore, the search for novel analgesic modalities for bone cancer pain has become urgent.

Nuclear factor of activated T cell (NFAT) is an important class of transcription factors. NFAT proteins are classified into five categories: NFAT1 (NFATc2 and NFATp), NFAT2 (NFATc1 and NFATc), NFAT3 (NFATc4), NFAT4 (NFATc3 and NFATx), and NFAT5 (TonEBP) [7]. Studies have shown that NFAT is not only expressed in T cells but also in various types of immune and nonimmune cells; it is crucial in regulating gene expression, cell cycle progression, embryonic development, and differentiation [8, 9]. With the exception of NFAT5, the activation of all NFATs is tightly controlled through dephosphorylation by the Ca^2+^‐activated protein phosphatase calmodulin phosphatase [10]. NFAT1 is consistently upregulated in microglia after spinal nerve ligation‐induced neuropathic pain [11]. However, whether and how NFAT1 regulates spinal microglia function and bone cancer pain have not been investigated.

IL‐18, a member of the IL‐1 family, is an important regulator of innate and acquired immune responses and has a crucial role in immune, inflammatory, tumor, and pain processes [12, 13, 14, 15, 16]. IL‐18 first binds to its specific ligand‐binding chain IL‐18 receptor to form a heterotrimeric complex [17]. The IL‐18R complex then recruits adaptive molecules, which induce the expression of proinflammatory cytokines, chemokines, or secondary mediators to participate in the inflammatory response [18, 19]. Studies have suggested that IL‐18 mediates communication between glia and neurons during neuropathic pain through interaction with IL‐18R, further contributing to the onset and progression of the inflammatory response [16, 20, 21]. IL‐18/IL‐18R signaling can modulate migraine progression through microglia–astrocyte crosstalk [22]. However, the role of IL‐18 signaling in bone cancer pain is unclear.

In this study, we used a Lewis lung carcinoma (LLC)‐induced bone cancer pain model in male mice to determine the expression and localization of NFAT1 in the spinal dorsal horn following tumor inoculation. We investigated the effects of NFAT1 on pain behavior in LLC‐induced bone cancer and on the release of the downstream proinflammatory cytokine IL‐18 through the p38 MAPK signaling pathway. Our findings could expand the understanding of the roles of NAFT1 signaling pathways in the spinal cord, particularly in relation to central sensitization and bone cancer pain hypersensitivity.

## 2 Materials and Methods

### 2.1 Animals

Adult male C57BL/6 mice (22 ± 2 g) were obtained from the Experimental Animal Center of Xuzhou Medical University. A specific pathogen‐free, temperature‐controlled facility was used to house the animals. The animals were under a 12‐h light–dark cycle and had free access to food and water. All animal experimental procedures were approved by the Xuzhou Medical University Animal Care and Use Committee. All procedures were performed according to the guidelines of the National Institutes of Health of China Guide for the Care and Use of Laboratory Animals.

### 2.2 Cell Culture

The mouse LLC cell line was purchased from Cell Bank/Stem Cell Bank, Chinese Academy of Sciences. The cells were seeded in Dulbecco's modified Eagle's medium (Gibco, Grand Island, NY, USA) containing 10% fetal bovine serum (Gibco) and 1% penicillin–streptomycin (Gibco). The cultures were maintained in a sterile cell incubator (Thermo, Waltham, MA, USA) at 37°C, with 5% CO_2_ and 95% O_2_. The cultures were passaged at a 1:2 ratio every 1–2 days.

### 2.3 Model of Bone Cancer Pain

LLC cells were detached using 0.125% trypsin, harvested through centrifugation, and resuspended at a concentration of 2 × 10^8^ cells/μL in sterile phosphate‐buffered saline (PBS). LLC cells were then injected into adult C57BL/6 male mice, as described previously [23]. In brief, mice were anesthetized with sodium pentobarbital (50 mg/kg) by administering intraperitoneal (i.p.) injection in the supine position. The right legs were shaved and disinfected with 75% (v/v) ethanol. A 30‐gauge needle was inserted into the right tibial medullary canal to create a pathway for injecting LLC cells. The 30‐gauge needle was then replaced with a 10‐μL Hamilton syringe. The 10‐μL volume containing approximately 2 × 10^6^ LLC cells was slowly injected in the bone cancer pain group, whereas an equivalent volume of sterile PBS was injected into the sham group. The needle was removed, and the injection hole was sealed with sterile bone wax to prevent cell leakage.

### 2.4 Drugs and Administration

The NFAT1 inhibitor 11R‐VIVIT (HY‐P1026) and p38 MAPK inhibitor SB239063 (HY‐11068) were purchased from MedChemExpress (USA). IL‐18 binding protein (IL‐18 BP, an IL‐18 antagonist, AF119) was purchased from R&D Systems (USA). 11R‐VIVIT and IL‐18 BP were dissolved in sterile PBS. SB239063 was dissolved in 5% Tween 80 and 5% DMSO in saline. All surgeries were done using anesthesia with sodium pentobarbital (50 mg/kg, i.p.). The reagents were administered intrathecally at a volume of 10 μL into the cerebral spinal fluid, with dosages determined through previous studies and preliminary experimentation [11, 24, 25]. The intrathecal injection procedure involved utilizing a 10‐μL Hamilton syringe equipped with a 30‐gauge needle, targeting the L5–L6 interspace. Needle insertion into the lumbar subarachnoid space was confirmed through a prompt tail and/or paw flick.

### 2.5 Bone Radiological Detection

Bone radiography was used to assess the extent of tibia bone destruction by LLC cells. Mice were placed on a transparent plane made of plexiglass and subjected to an X‐ray source under sodium pentobarbital anesthesia. Lateral radiographic images of the tibia were obtained using a digital radiography system (DR‐F, GE, Atlanta, GA, USA).

### 2.6 Bone Histological Detection

The bones were postfixed using 4% paraformaldehyde for 72 h and decalcified using 10% EDTA (pH 7.4) for 3 weeks. The tibias were then washed, dehydrated, paraffin‐embedded, sliced into 5‐μm sections, and stained with hematoxylin and eosin to examine the invasion of carcinoma cells and the destruction of bone tissue.

### 2.7 Mechanical Allodynia

The paw withdrawal threshold (PWT) was measured as an index of mechanical allodynia using von Frey filaments (Aesthesio, Danmic Global, San Jose, CA, USA) [26]. All experiments were conducted in a polymethyl methacrylate box with a wire mesh flooring. In brief, mice were acclimated to the experimental setting for three consecutive days, followed by an additional 20‐min acclimation immediately prior to testing. Filaments weighing 0.02, 0.04, 0.07, 0.4, 0.6, 1.0, and 1.4 g were applied to the midplantar surface of the hind paw with sufficient pressure to either elicit a bending response lasting 3–4 s or provoke a withdrawal reflex, flinching or licking within 4 s. Finally, 50% PWT was determined using Dixon's up‐and‐down method.

### 2.8 Thermal Hyperalgesia

The paw withdrawal latency (PWL) was tested to assess thermal hyperalgesia using a Plantar Analgesia Meter (Model 390, Series 8; IITC Life Science, USA) [27]. Prior to the experiment, the mice were acclimatized for 3 days consecutively. Subsequently, the mice were placed in a plexiglass chamber on a glass plate and acclimatized for 10–15 min before commencing the experiment. Latency (s) was defined as the duration from the initiation of the radiant heat stimulus until the withdrawal of the hind paw, and a 20‐s cut‐off was established to prevent any potential harm to the tissue. To determine latency, the heat stimulus was repeated three times with a 10‐min interval between each repetition [28].

### 2.9 Spontaneous Pain

The occurrence of spontaneous pain in the hindlimbs was identified as described previously [29]. In detail, plexiglass compartments, measuring 10 × 10 × 15 cm, were positioned on a raised glass platform. The spontaneous lifting of the right hind paw, characterized by a swift elevation of the entire limb (commencing with hip flexion and encompassing dorsiflexion of the toes) and not associated with locomotion or grooming was considered a single flinch. The animals were observed three times, each for 10 min with a 5‐min break.

### 2.10 Limb‐Use Score

The mice were positioned on a glass plate and observed during spontaneous ambulation. A scoring system was employed to assess locomotor abilities as follows: scores 0, 1, 2, 3, and 4 indicated complete absence of limb usage, partial loss of limb usage, evident limping and guarding, slight limping and guarding, and normal walking, respectively [3].

### 2.11 Real‐Time Quantitative Polymerase Chain Reaction (RT‐qPCR)

Total RNA was extracted from L4–6 spinal dorsal horn tissues using TRIzol reagent (Invitrogen, USA) following the manufacturer's instructions. Subsequently, 1 μg of the extracted total RNA was reverse transcribed into cDNA employing PrimeScript RT Master Mix (RR036A; Takara, Japan). Each experimental procedure was performed in triplicate using a 20‐μL reaction mixture containing 2 μL of cDNA and 10 μM of gene‐specific primers. SYBR Premix Ex Taq Kit (RR420A; Takara) was used, and the reactions were conducted on a 7300 Plus Real‐Time PCR System (Applied Biosystems, USA). The thermocycling conditions included an initial denaturation step at 95°C for 30 s, followed by 40 amplification cycles consisting of 5 s at 95°C and 30 s at 60°C. The mRNA levels were assessed utilizing the 2^−∆∆Ct^ method, with *GAPDH* as an endogenous control. The following primer pairs were used for RT‐qPCR: *NFAT1* primer forward: 5′‐CGC ACG CCT TCT ACC AAG TA‐3′, *NFAT1* primer reverse: 5′‐GTC GAT GGT GGC TCT CAT GT‐3′ and *GAPDH* primer forward: 5′‐AAA TGG TGA AGG TCG GTG TG‐3′, *GAPDH* primer reverse: 5′‐AGG TCA ATG AAG GGG TCG TT‐3′.

### 2.12 Western Blotting

Western blotting was performed as described previously [3]. Briefly, whole proteins from L4–L6 spinal cord segments were extracted using lysis buffer with protease and phosphatase inhibitor cocktails. Protein concentrations were determined using the BCA Protein Assay (Beyotime, P0010, China). In total, 30 μg of protein was separated using SDS‐PAGE and transferred to a PVDF membrane (Merck Millipore, ISEQ00010, USA). The membranes were blocked with 5% milk and incubated overnight at 4°C with the following primary antibodies: rabbit anti‐NFAT1 (1:000, Cell Signaling Technology, catalog #4389S), rabbit anti‐p‐p38 (1:000, Cell Signaling Technology, catalog #4511), rabbit anti‐c‐Fos (1:1000, Cell signaling technology, catalog #2250), goat anti‐IBA‐1 (1:500, Abcam, catalog #ab5076), goat anti‐IL‐18 (1:500, R&D Systems, catalog #AF521), rabbit anti‐p‐NR2B (1:1000, Cell Signaling Technology, catalog #4208), rabbit anti‐p‐CaMKIl (1:1000, Cell Signaling Technology, catalog #12716), rabbit anti‐p‐CREB (1:1000, Cell Signaling Technology, catalog #9198), and mouse anti‐GAPDH (1:10000, Sigma‐Aldrich, catalog #MAB374). The PVDF membranes were washed with TBST three times (10 min each), incubated at room temperature for 1 h with the corresponding horseradish peroxidase‐conjugated secondary antibodies (1:2000, Beyotime) diluted in TBST, and washed with TBST three times (10 min each). Signals were detected using an enhanced chemiluminescence detection system (Beyotime) and revealed by the Alliance Q9 Imager (Uvitec). Band intensity was quantified using ImageJ software.

### 2.13 Immunofluorescence

Mice were anesthetized deeply and perfused through the ascending aorta with 0.9% saline followed by 4% paraformaldehyde in 0.1 M phosphate buffer (pH 7.4). After perfusion was completed, the L4–L6 spinal cord segment was removed and postfixed in the same fixative overnight and cryoprotected in 30% sucrose. Spinal cord sections (30 μm, free‐floating) were prepared with a freezing microtome (VT1000S, Leica Microsystems), blocked with 5% donkey serum albumin for 2 h at room temperature, and incubated overnight at 4°C with the primary antibody. The following primary antibodies were used: rabbit anti‐NFAT1 (1:500, Cell Signaling Technology, catalog #4389S), mouse anti‐NFAT1 (1:500, Abcam, catalog #Ab2722), mouse anti‐NeuN (1:500, Sigma‐Aldrich, catalog #MAB377), mouse anti‐GFAP (1:500, Sigma‐Aldrich, catalog #MAB360), goat anti‐IBA‐1 (1:500, Abcam, catalog #ab5076), rabbit anti‐IBA‐1 (1:500, Wako, catalog #019‐19741), rabbit anti‐c‐Fos (1:500, Cell signaling technology, catalog #2250), rabbit anti‐p‐p38 (1:500, Cell Signaling Technology, catalog #4511), goat anti‐IL‐18 (1:500, R&D Systems, catalog #AF521), goat anti‐IL‐18R (1:500, R&D Systems, catalog #AF856), and rabbit anti‐NR2B (1:500, Abcam, catalog #ab65783). The sections were washed, incubated with Alexa Fluor 488‐ or 594‐conjugated secondary antibodies (1:500; Invitrogen) in the dark at room temperature for 2 h, and mounted using ProLong Gold Antifade Reagent with DAPI (Abcam, ab104139). Fluorescence intensity was visualized under a confocal microscope (FluoView1000; Olympus). Quantitative analyses were calculated using ImageJ software.

### 2.14 Statistical Analysis

Data are presented as the mean ± SEM. All data from different groups were verified for normality and homogeneity of variance using Kolmogorov–Smirnov and Brown–Forsythe tests before analysis. For pain behavioral experiments, differences between groups for threshold and latency results were determined using two‐way analysis of variance (ANOVA) with the post hoc Bonferroni test for multiple comparisons. Differences between groups for spontaneous pain and limb‐use score results were determined using one‐way ANOVA with post hoc Dunnett's test for multiple comparisons. For RT‐qPCR, western blotting, and immunofluorescence analysis, group differences were determined using one‐way ANOVA with post hoc Dunnett's test for multiple comparisons. Differences were considered statistically significant when *p* values were < 0.05 (Tables S1 and S2). All statistical analyses were performed using GraphPad Prism 8.0 software.

## 3 Results

### 3.1 Intratibial LLC Inoculation Induces Bone Destruction and Pain Hypersensitivity

**FIGURE 1:**
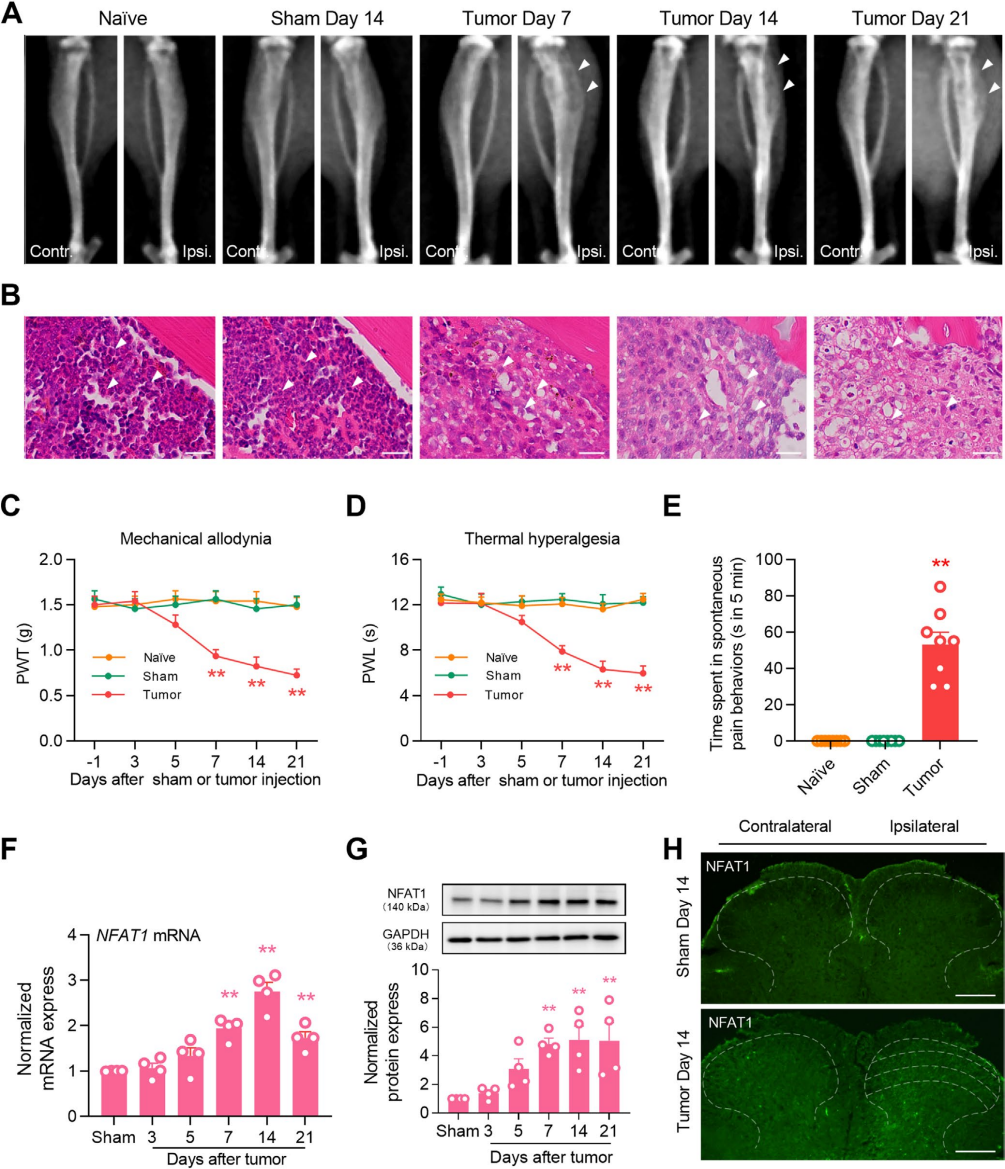
Lewis lung carcinoma inoculation induces bone destruction and pain hypersensitivity, as well as NFAT1 expression in the spinal cord. (A) Radiological results demonstrated signs of osteolytic destruction in the proximal epiphysis of the ipsilateral tibia (white arrow) in a time‐dependent manner. Significant changes were not observed in naïve and sham (14 days following sham operation) mice. (B) Hematoxylin and eosin staining showed that bone substance and bone marrow were destroyed and infiltrated by cancer cells (white arrow) after Lewis lung carcinoma inoculation. Scale bars: 25 μm. (C, D) Behavioral analysis showed that Lewis lung carcinoma inoculation induced reduction of paw withdrawal threshold (PWT) (C) and paw withdrawal latency (PWL) (D) in the affected hind paw. Data are expressed as the mean ± SEM. **p < 0.01 vsersus naïve group, *N* = 8 for each group, two‐way repeated‐measures ANOVA with the post hoc Bonferroni test. (E) Tumor inoculation induced a significant increase in spontaneous hindlimb lifting in the affected hind paw on day 14 after tumor induction. Data are expressed as the mean ± SEM. **p < 0.01 versus naïve group, *N* = 8 for each group, one‐way ANOVA with post hoc Dunnett's test. (F) Real‐time quantitative polymerase chain reaction analysis for the time course changes of *NFAT1* mRNA in mice before and after tumor inoculation. Data were normalized to the housekeeping gene *GAPDH*. (G) Western blot analysis for the time course changes of NFAT1 expression in mice before and after tumor inoculation. Data were normalized to the housekeeping protein GAPDH. (F, G) Data are expressed as the mean ± SEM. **p < 0.01 versus sham group. *N* = 4 for each time point, one‐way ANOVA with post hoc Dunnett's test. (H) Immunofluorescence images of NFAT1 immunoreactivity in the spinal dorsal horn of mice. Tissues were collected on day 14 after sham or tumor inoculation. Scale bars: 100 μm.

To confirm the establishment of the bone cancer pain model in mice, we assessed the microscopic features of tumor progression and bone destruction within the bone microenvironment after LLC inoculation. On day 14, the ipsilateral tibial bone marrow was not destroyed, the bone cortex was smooth, and bone tumor growth was not detected in sham mice compared with that in naïve mice (Figure 1A). By contrast, 7 days after tumor inoculation, osteolytic destruction was detected in the proximal epiphysis of the ipsilateral tibial bone, specifically in close proximity to the site of LLC injection. At 14 days after tumor inoculation, bone destruction notably increased at the epiphyseal region of the tibia. The destruction of trabecular structures was exacerbated, accompanied by the deteriorating medullary and cortical bone loss. Tumor cells migrated from the tibial cavity into the surrounding soft tissues on day 21 (Figure 1A). Additionally, hematoxylin and eosin staining showed bone destruction in the ipsilateral tibia, manifesting as the time‐dependent replacement of bone marrow cells by carcinoma cells and disappearance of healthy trabecular structures (Figure 1B). Consistent with previous studies [23], mice with tumors experienced increasing sensitivity to pain after tumor injection. The PWT and PWL were significantly decreased from day 7 to day 21 after tumor injection (Figure 1C,D). On day 14 post‐tumor inoculation, spontaneous hindlimb lifting increased significantly (Figure 1E).

### 3.2 NFAT1 Is Upregulated in the Spinal Cord After Tumor Inoculation

**FIGURE 2:**
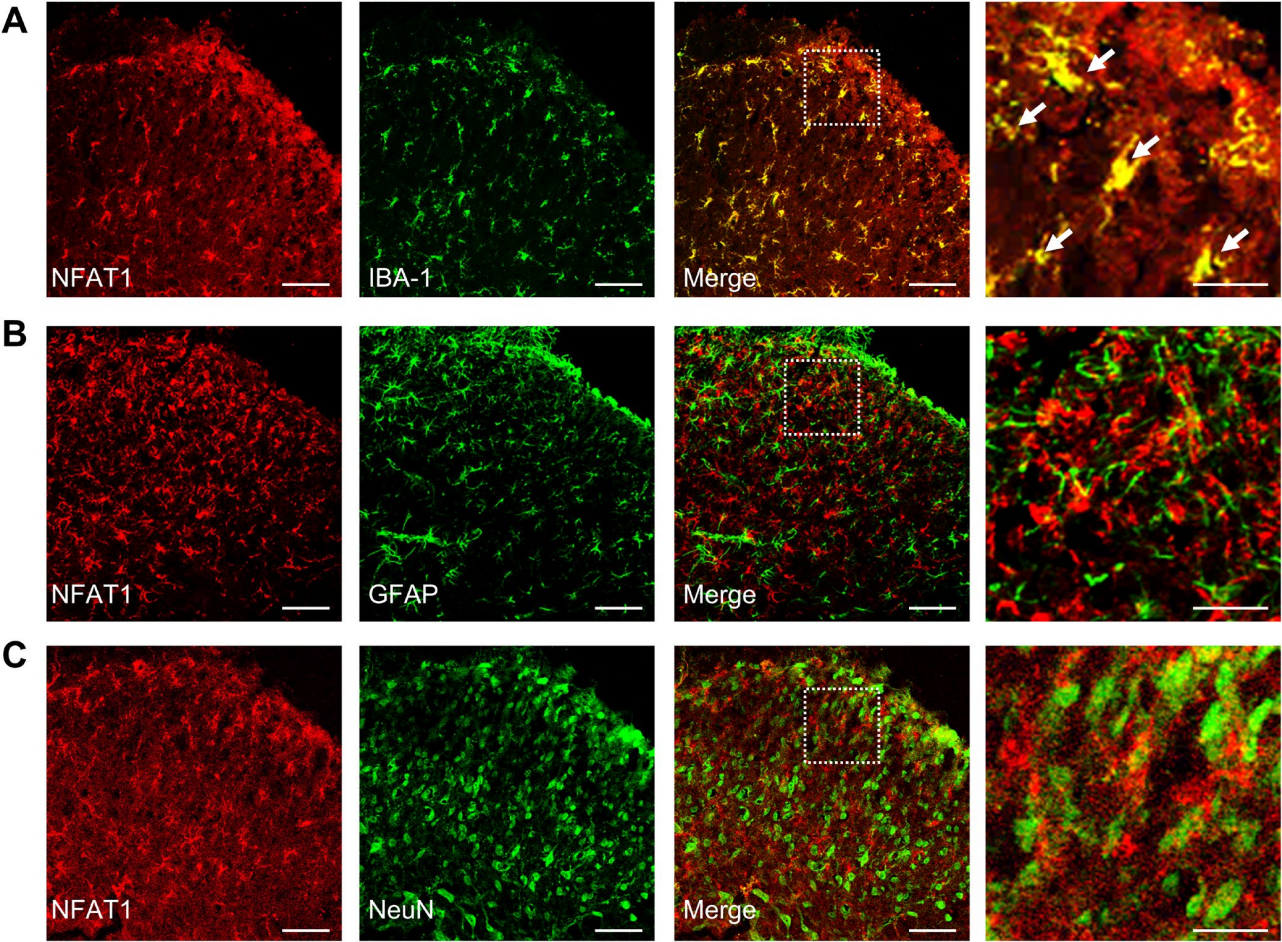
Cellular localization of NFAT1 in the spinal cord after tumor inoculation. (A–C) Immunofluorescence staining of NFAT1 combined with cell markers in the spinal dorsal horn: Ionized calcium‐binding adaptor molecule 1 (IBA‐1; microglia) (A), glial fibrillary acidic protein (GFAP; astrocytes) (B), and neuronal nuclei (NeuN; neurons) (C). Tissues were collected on day 14 post‐tumor inoculation. Scale bars: 50 μm and 20 μm (zoom).

To evaluate the expression and distribution of NFAT1 in the spinal dorsal horn after LLC inoculation, we first analyzed changes in the mRNA expression of *NFAT1* in bone cancer pain. Compared to that in sham mice, in mice inoculated with tumor *NFAT1* mRNA was substantially upregulated in the spinal dorsal horn on day 7, persisting until day 21 post‐tumor inoculation (Figure 1F). Consistent with RT‐qPCR analysis, western blot analysis revealed that tumor inoculation led to a sustained increase in NFAT1 protein expression in the spinal dorsal horn. This increase was significant by day 7 and continued through day 21 (Figure 1G). Furthermore, immunofluorescence revealed that the increased expression of NFAT1 was distributed predominately in the ipsilateral, but not contralateral, spinal dorsal horn on day 14 post‐tumor inoculation (Figure 1H). We further examined the cellular localization of NFAT1 via immunostaining with various cell markers in the spinal dorsal horn on day 14 post‐tumor inoculation. Immunofluorescence demonstrated that NFAT1 predominantly colocalized with the microglial marker IBA‐1 (Figure 2A), but not with the astrocytic marker GFAP (Figure 2B) or neuronal marker NeuN (Figure 2C). Taken together, these results suggest that spinal NFAT1 is functionally upregulated after tumor inoculation.

### 3.3 NFAT1 Mediates Bone Cancer Pain Behaviors

**FIGURE 3:**
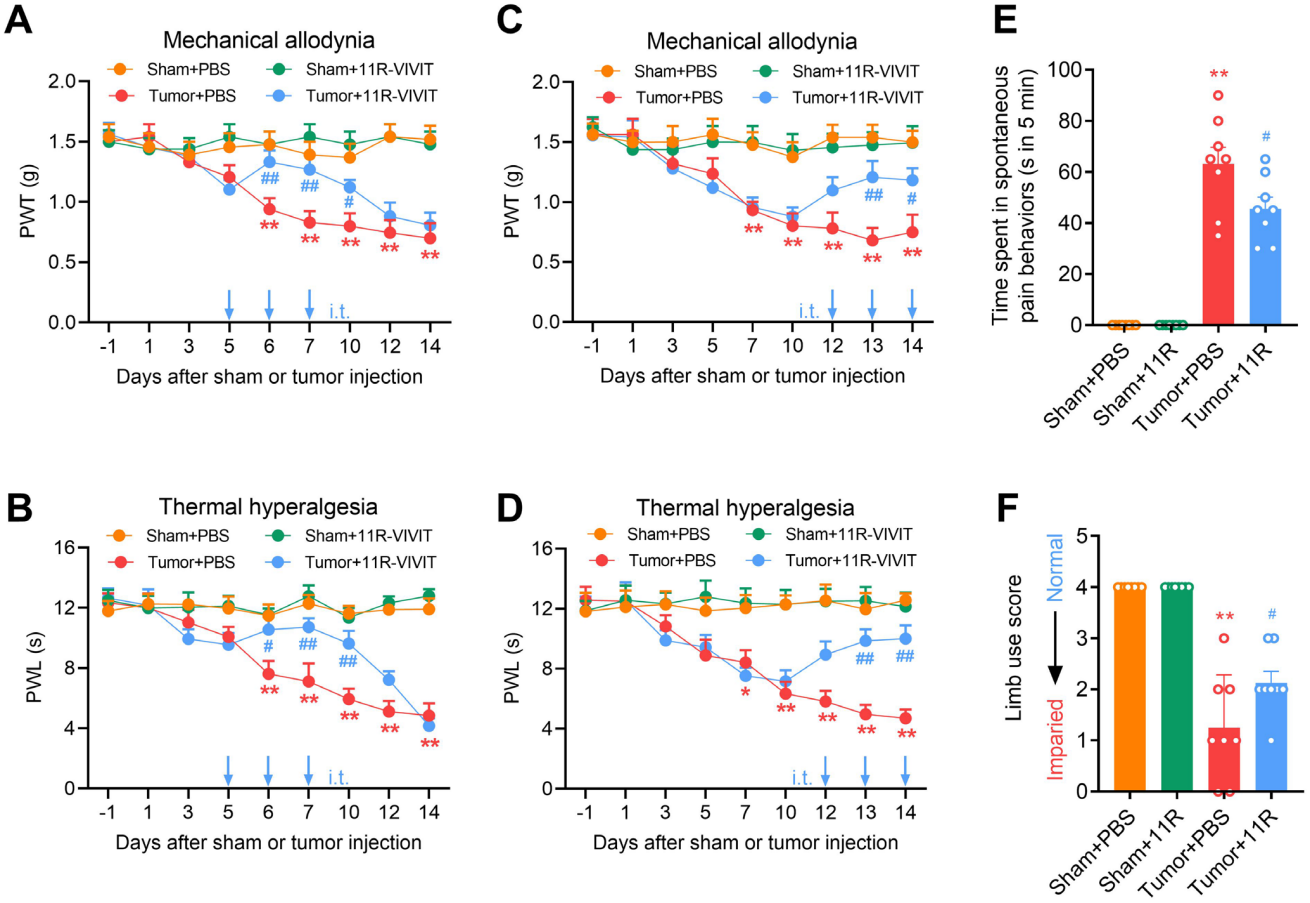
Pharmacological inhibition of NFAT1 prevents and attenuates mechanical allodynia, thermal hyperalgesia and spontaneous pain in tumor‐bearing mice. (A–D) Repeated administration of the NFAT1 inhibitor 11R‐VIVIT significantly delayed or attenuated tumor‐induced reduction in paw withdrawal threshold (PWT) (A, C) and paw withdrawal latency (PWL) (B, D) in tumor‐bearing mice. 11R‐VIVIT (1 nM/10 μL, i.t.) or PBS (vehicle control, 10 μL, i.t.) was administered once daily on days 5, 6, and 7 (A, B) or days 12, 13, and 14 (C, D) post‐tumor inoculation. Behavioral tests were performed 4 h after each injection. Data are expressed as the mean ± SEM. *p < 0.05, **p < 0.01 versus sham + PBS group; #*p* < 0.05, ##*p* < 0.01 versus tumor + PBS group. *N* = 8 for each group, two‐way repeated‐measures ANOVA with the post hoc Bonferroni test. (E, F) Repeated administration of the NFAT1 inhibitor 11R‐VIVIT significantly attenuated tumor‐induced spontaneous pain behaviors (E) and limb uselessness (F) in tumor‐bearing mice. 11R‐VIVIT (1 nM/10 μL, i.t.) or PBS (vehicle control, 10 μL, i.t.) was administered once daily on days 12, 13, and 14 post‐tumor inoculation. Behavioral tests were performed 4 h after the last injection on day 14. Data are expressed as the mean ± SEM. **p < 0.01 versus sham + PBS group; #*p* < 0.05 versus tumor + PBS group. *N* = 8 for each group, one‐way ANOVA with post hoc Dunnett's test.

Next, we employed pharmacologic approaches to examine the regulatory role of NFAT1 in the production and maintenance of bone cancer pain. Repeated spinal administration of the NFAT1‐specific inhibitor 11R‐VIVIT once daily on days 5, 6, and 7 post‐tumor inoculation (early‐phase treatment) significantly delayed the onset of mechanical allodynia (Figure 3A) and thermal hyperalgesia (Figure 3B). Furthermore, repeated intrathecal injections of 11R‐VIVIT once daily on days 12, 13, and 14 post‐tumor inoculation (late‐phase treatment) effectively and persistently attenuated tumor‐induced mechanical allodynia (Figure 3C) and thermal hyperalgesia (Figure 3D). Tumor‐related spontaneous pain (Figure 3E) and limb uselessness (Figure 3F) were relieved by repeated intrathecal injections of 11R‐VIVIT on day 14 post‐tumor inoculation. Collectively, these behavioral data suggest that spinal NFAT1 contributes to bone cancer pain.

### 3.4 NFAT1 Contributes to Tumor Inoculation‐Induced Central Sensitization

**FIGURE 4:**
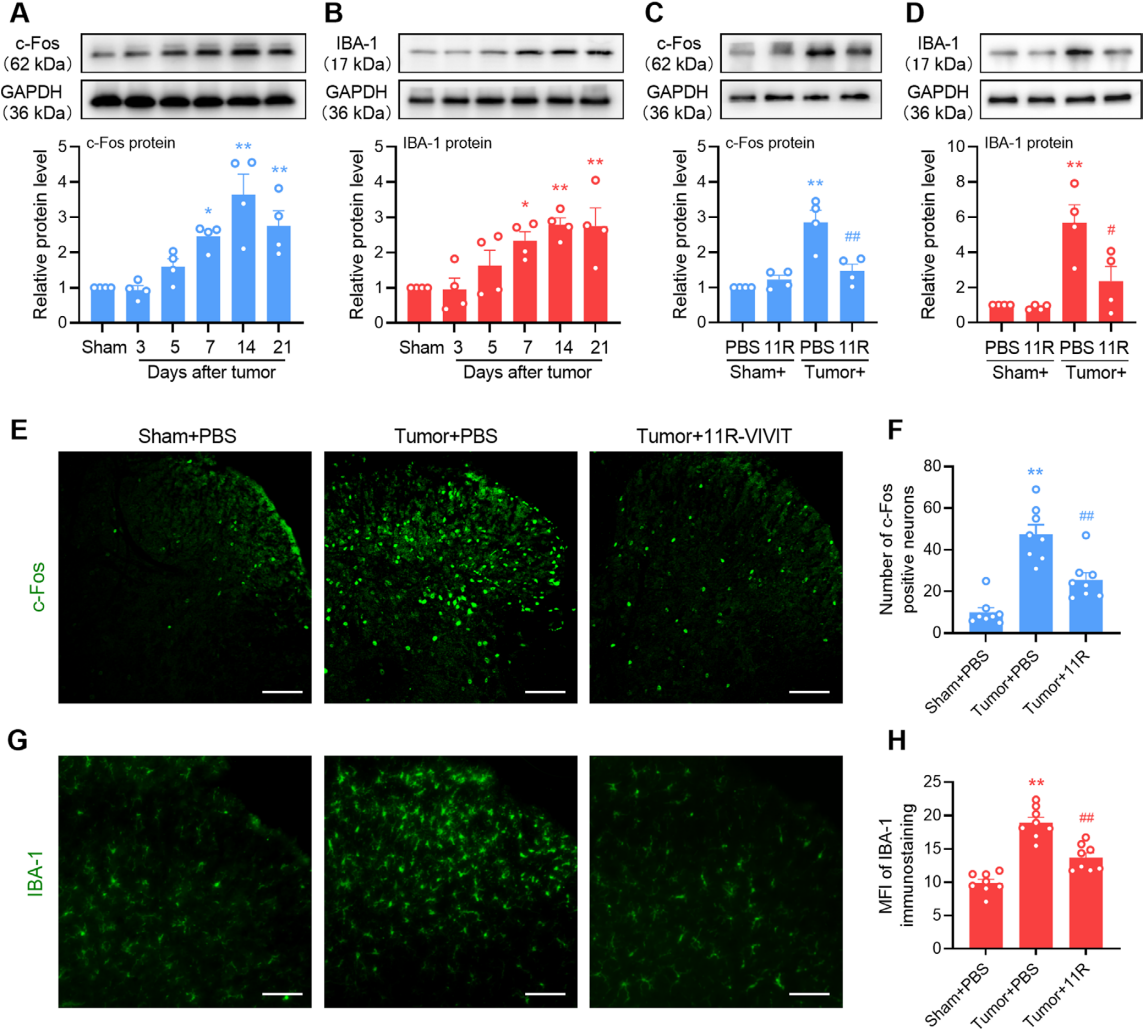
Pharmacological inhibition of NFAT1 suppresses tumor‐induced neuronal sensitization and microglial activation. (A, B) Western blot analysis for the time course changes of c‐Fos (A) and IBA‐1 (B) expression in mice before and after tumor inoculation. Data are expressed as the mean ± SEM. *p < 0.05, **p < 0.01 versus sham group. *N* = 4 for each time point, one‐way ANOVA with post hoc Dunnett's test. (C, D) Effects of repeated administration of the NFAT1 inhibitor 11R‐VIVIT on tumor‐induced upregulation of c‐Fos (C) and IBA‐1 (D) expression in the spinal dorsal horn. 11R‐VIVIT (1 nM/10 μL, i.t.) or PBS (vehicle control, 10 μL, i.t.) was injected once daily on days 12, 13, and 14 post‐tumor inoculation. Tissues were collected 4 h after the last injection on day 14. Data are expressed as the mean ± SEM. *p < 0.05, **p < 0.01 versus sham + PBS group; #*p* < 0.05, ##*p* < 0.01 versus tumor + PBS group. *N* = 4 for each group, one‐way ANOVA with post hoc Dunnett's test. (E–H) Effects of repeated administration of the NFAT1 inhibitor 11R‐VIVIT on the tumor‐increased number of c‐Fos (E, F) and mean fluorescence intensity (MFI) of IBA‐1 (G, H). 11R‐VIVIT (1 nM/10 μL, i.t.) or PBS (vehicle control, 10 μL, i.t.) was injected once daily on days 12, 13, and 14 post‐tumor inoculation. Tissues were collected 4 h after the last injection on day 14. Data are expressed as the mean ± SEM. **p < 0.01 versus sham + PBS group; ##*p* < 0.01 versus tumor + PBS group. *N* = 8 spinal slices, one‐way ANOVA with post hoc Dunnett's test. Scale bars: 50 μm.

Because NFAT1 was expressed and increased in the spinal microglia, we investigated whether NFAT1 is involved in neuronal sensitization and microglial activation following tumor inoculation. Western blot analysis revealed that tumor inoculation induced the upregulation of c‐Fos (Figure 4A) and IBA‐1 (Figure 4B) in a time‐dependent manner, indicating neuronal sensitization and microglial activation in the spinal dorsal horn following tumor inoculation, respectively. However, repeated intrathecal injections of 11R‐VIVIT significantly suppressed the tumor‐induced upregulation of c‐Fos (Figure 4C) and IBA‐1 (Figure 4D). Immunofluorescence analysis revealed that repeated injections of 11R‐VIVIT significantly suppressed the tumor‐induced increase in the number of c‐Fos‐positive neurons (Figure 4E,F) and mean fluorescence intensity of IBA‐1‐labeled microglia (Figure 4G,H) in the spinal dorsal horn. Taken together, spinal NFAT1 may directly affect the sensitization of neurons and the activation of microglia in bone cancer pain.

### 3.5 NFAT1 Activates Microglial p38 Pathways in the Spinal Cord After Tumor Inoculation

**FIGURE 5:**
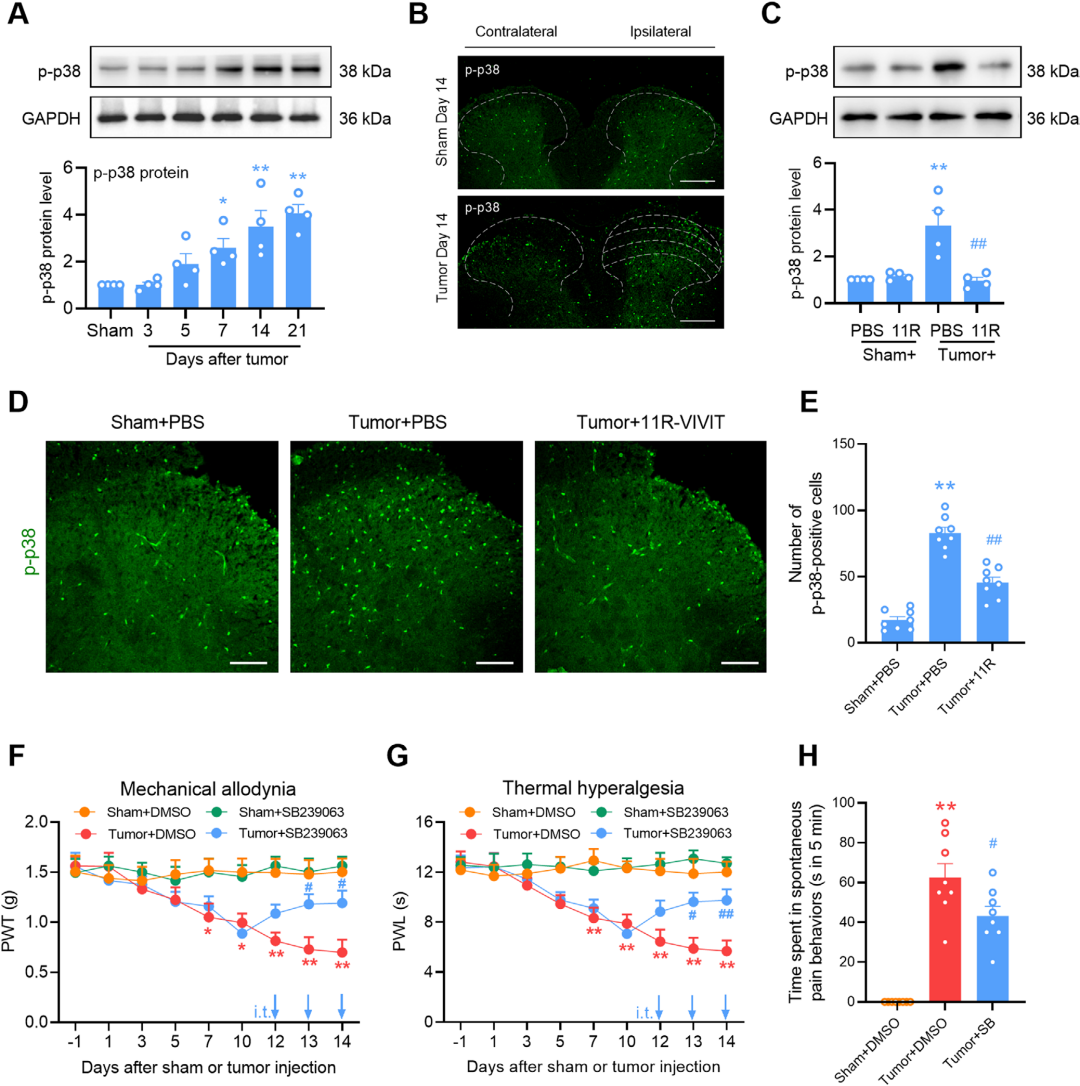
p‐p38 MAPK signals are activated by NFAT1 after tumor inoculation. (A) Tumor inoculation induced the upregulation of p‐p38 in a time‐dependent manner. Data are expressed as the mean ± SEM. *p < 0.05, **p < 0.01 versus sham group. *N* = 4 for each time point, one‐way ANOVA with post hoc Dunnett's test. (B) Immunofluorescence showed that p‐p38 MAPK was low in both sides of the spinal dorsal horn in sham mice; but increased in the ipsilateral spinal dorsal horn compared with the contralateral spinal dorsal horn in tumor‐bearing mice. Tissues were collected on day 14 after sham or tumor inoculation. Scale bars: 100 μm. (C) Effects of repeated administration of the NFAT1 inhibitor 11R‐VIVIT on the tumor‐induced upregulation of p‐p38 MAPK expression in the spinal dorsal horn. 11R‐VIVIT (1 nM/10 μL, i.t.) or PBS (vehicle control, 10 μL, i.t.) was injected once daily on days 12, 13, and 14 post‐tumor inoculation. Tissues were collected 4 h after the last injection on day 14. Data are expressed as the mean ± SEM. **p < 0.01 versus sham + PBS group; ##*p* < 0.01 versus tumor + PBS group. *N* = 4 for each group, one‐way ANOVA with post hoc Dunnett's test. (D, E) Effects of repeated administration of the NFAT1 inhibitor 11R‐VIVIT on the tumor‐induced increase in the number of p‐p38 MAPK. 11R‐VIVIT (1 nM/10 μL, i.t.) or PBS (vehicle control, 10 μL, i.t.) was injected once daily on days 12, 13, and 14 post‐tumor inoculation. Tissues were collected 4 h after the last injection on day 14. Data are expressed as the mean ± SEM. **p < 0.01 versus sham + PBS group; ##*p* < 0.01 versus tumor + PBS group. *N* = 8 spinal slices, one‐way ANOVA with post hoc Dunnett's test. Scale bars: 50 μm. (F, G) Effects of repeated administration of the p‐p38 inhibitor SB239063 on tumor‐induced mechanical allodynia (F) and thermal hyperalgesia (G). SB239063 (2 μg/10 μL, i.t.) or DMSO (5%, 10 μL, i.t.) was injected once daily on days 12, 13, and 14 post‐tumor inoculation. Behavioral tests were performed 4 h after each injection. Data are expressed as the mean ± SEM. *p < 0.05, **p < 0.01 versus sham + DMSO group; #*p* < 0.05, ##*p* < 0.01 versus tumor + DMSO group. *N* = 8 for each group, two‐way repeated‐measures ANOVA with the post hoc Bonferroni test. (H) Effects of repeated administration of p‐p38 inhibitor SB239063 on tumor‐induced spontaneous pain. SB239063 (2 μg/10 μL, i.t.) or DMSO (5%, 10 μL, i.t.) was administered once daily on days 12, 13, and 14 post‐tumor inoculation. Behavioral tests were performed 4 h after the last injection on day 14. Data are expressed as the mean ± SEM. **p < 0.01 versus sham + DMSO group; #*p* < 0.05 versus tumor + DMSO group. *N* = 8 for each group, one‐way ANOVA with post hoc Dunnett's test.

**FIGURE 6:**
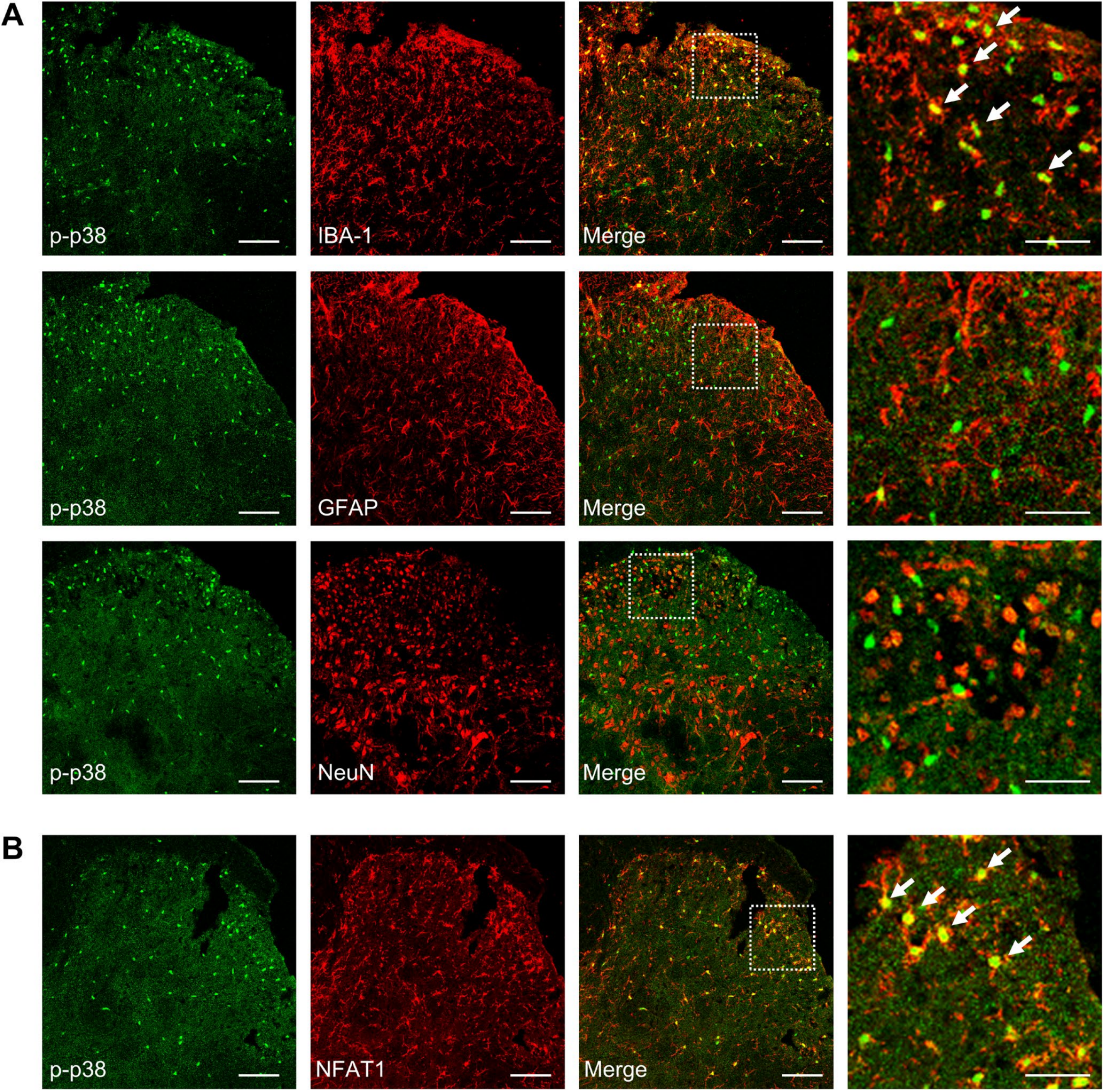
Cellular localization of p‐p38 MAPK in the spinal cord after tumor inoculation. (A) p‐p38 MAPK immunoreactivity colocalized with IBA‐1, but not with GFAP or NeuN in the spinal dorsal horn. (B) p‐p38 MAPK immunoreactivity colocalized with NFAT1 in the spinal dorsal horn. Tissues were collected on day 14 post‐tumor inoculation. Scale bars: 50 and 20 μm (zoom).

The P38 MAPK pathway, which is mainly distributed in the spinal microglia, plays an important role in neuroinflammation and neuropathic pain [30]. Also, it is reported that p38 MAPK is a critical signaling transducer in the NFAT1 signaling pathway [11, 31]. We evaluated whether the p38 MAPK signaling pathway was downstream of NFAT1 after tumor inoculation in the spinal cord. Compared to sham mice, mice inoculated with tumor had an upregulation of phosphorylated p38 (p‐p38) in a time‐dependent manner in the spinal dorsal horn, which commenced on day 7 and was maintained at a high level until day 21 (Figure 5A). Consistently, immunostaining showed a marked increase in p‐p38 in the ipsilateral spinal cord on day 14 post‐tumor inoculation compared with the contralateral side or sham control (Figure 5B). However, the tumor‐induced upregulation of p‐p38 was reduced by the repeated intrathecal injections of 11R‐VIVIT (Figure 5C). The effect of NFAT1 on p‐p38 upregulation was further confirmed via immunofluorescence analysis (Figure 5D,E). Repeated intrathecal injections of the p38 inhibitor SB239063 once daily on days 12, 13, and 14 post‐tumor inoculation attenuated tumor‐induced mechanical allodynia (Figure 5F) and thermal hyperalgesia (Figure 5G). Tumor‐related spontaneous pain (Figure 5H) was relieved by repeated intrathecal injections of SB239063 on day 14 post‐tumor inoculation. Immunofluorescence staining confirmed that p‐p38 predominantly colocalized with IBA‐1 (Figure 6A) and NFAT1 (Figure 6B) but not with NeuN or GFAP (Figure 6A) in the spinal dorsal horn on day 14 post‐tumor inoculation. Thus, p38 MAPK could be downstream of NFAT1 in spinal microglia after tumor inoculation.

### 3.6 NFAT1 Mediates Tumor‐Induced IL‐18 Upregulation in Spinal Microglia via p38 MAPK

**FIGURE 7:**
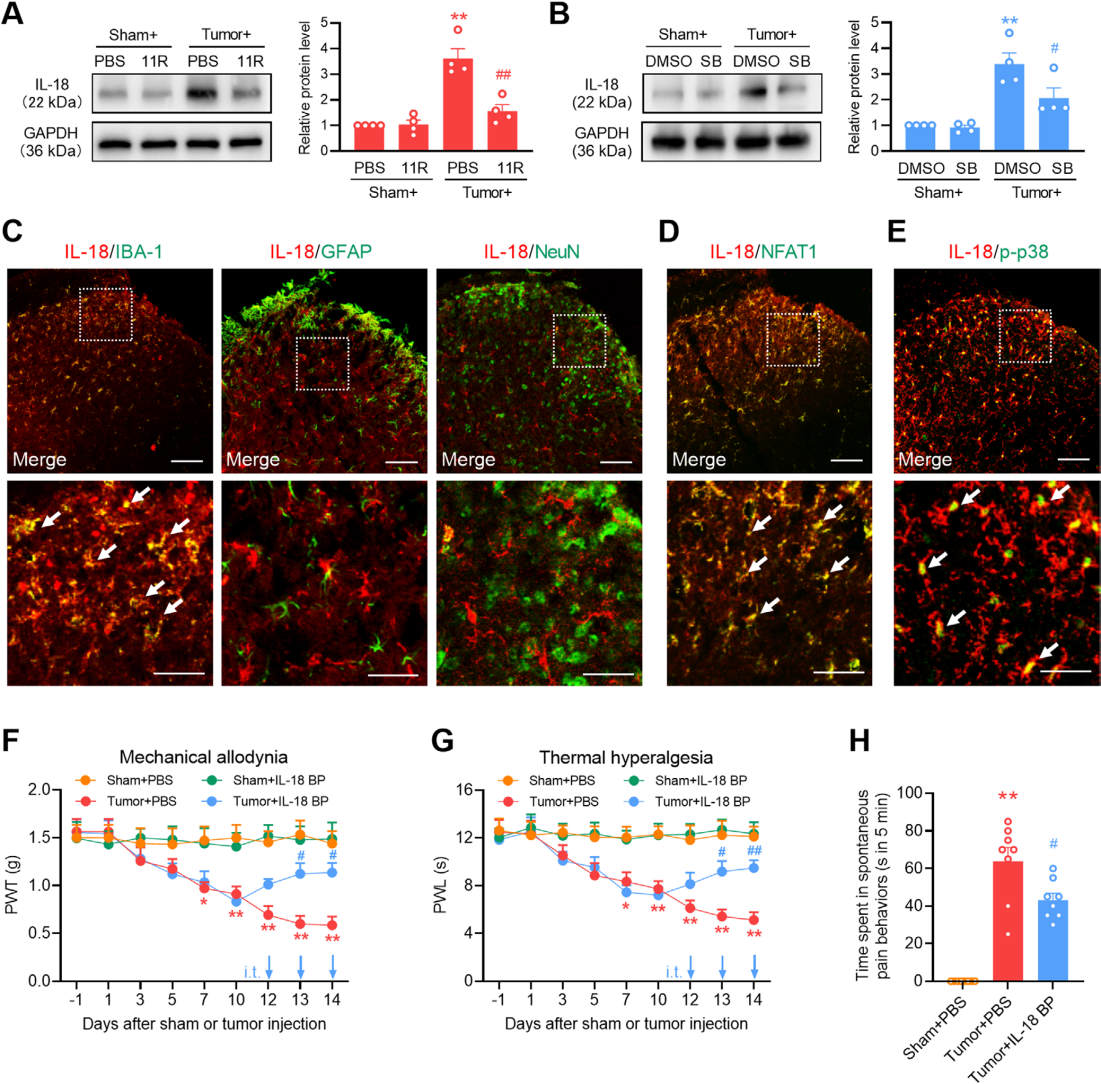
Spinal blockade of the NFAT1/p38 pathway suppresses tumor‐induced IL‐18 production. (A, B) Western blot analysis showed that 11R‐VIVIT (A), SB239063 (B) significantly suppressed the tumor‐induced upregulation of IL‐18 expression in the spinal dorsal horn. 11R‐VIVIT (1 nM/10 μL, i.t.), PBS (vehicle control, 10 μL, i.t.), SB239063 (2 μg/10 μL, i.t.), or DMSO (5%, 10 μL, i.t.) was administered once daily on days 12, 13, and 14 post‐tumor inoculation. Tissues were collected 4 h after the last injection on day 14. Data are expressed as the mean ± SEM. **p < 0.01 versus sham + PBS (or DMSO) group; #*p* < 0.05, ##*p* < 0.01 versus tumor + PBS (or DMSO) group. *N* = 4 for each group, one‐way ANOVA with post hoc Dunnett's test. (C) IL‐18 immunoreactivity colocalized with IBA‐1 but not with GFAP or NeuN in the spinal dorsal horn. (D, E) IL‐18 immunoreactivity colocalized with NFAT1 (D) and p‐p38 MAPK (E) in the spinal dorsal horn. Tissues were collected on day 14 post‐tumor inoculation. Scale bars: 50 μm and 20 μm (zoom). (F, G) Repeated administration of IL‐18 BP significantly attenuated tumor‐induced reduction in the paw withdrawal threshold (PWT) (F) and paw withdrawal latency (PWL) (G) in tumor‐bearing mice. IL‐18 BP (1 μg/10 μL, i.t.) or PBS (vehicle control, 10 μL, i.t.) was injected once daily on days 12, 13, and 14 post‐tumor inoculation. Behavioral tests were performed 4 h after each injection. Data are expressed as the mean ± SEM. *p < 0.05, **p < 0.01 versus sham + PBS group; #*p* < 0.05, ##*p* < 0.01 versus tumor + PBS group. *N* = 8 for each group, two‐way repeated‐measures ANOVA with the post hoc Bonferroni test. (H) Repeated administration of IL‐18 BP significantly attenuated tumor‐induced spontaneous pain behaviors in tumor‐bearing mice. IL‐18 BP (1 μg/10 μL, i.t.) or PBS (vehicle control, 10 μL, i.t.) was administered once daily on days 12, 13, and 14 post‐tumor inoculation. Behavioral tests were performed 4 h after the last injection on day 14. Data are expressed as the mean ± SEM. **p < 0.01 versus sham + PBS group; #*p* < 0.05 versus tumor + PBS group. *N* = 8 for each group, one‐way ANOVA with post hoc Dunnett's test.

Under various pain conditions, reactive microglial cells in the spinal cord promote the persistent production of proinflammatory cytokines [30], which in turn promote inflammation in glial cells and affect synaptic plasticity and neuronal excitability [32]. To evaluate the microglial mechanism of NFAT1/p38 in bone cancer pain, we examined changes in the expression of the proinflammatory cytokine IL‐18 in the spinal dorsal horn through the spinal blockade of NFAT1 or p38. Western blot analysis revealed a pronounced increase in IL‐18 expression on day 14 post‐tumor inoculation. However, intrathecal administration of 11R‐VIVIT (Figure 7A) or SB239063 (Figure 7B) reduced tumor‐induced IL‐18 expression. Immunofluorescence staining confirmed that IL‐18 was predominantly expressed in the microglia (Figure 7C) but not in the neurons or astrocytes. IL‐18 colocalized with NFAT1 (Figure 7D) and p‐p38 (Figure 7E) in the spinal dorsal horn on day 14 post‐tumor inoculation. Repeated intrathecal injections of the IL‐18 inhibitor IL‐18 BP once daily on days 12, 13, and 14 post‐tumor inoculation attenuated tumor‐induced mechanical allodynia (Figure 7F), thermal hyperalgesia (Figure 7G), and spontaneous pain (Figure 7H). These findings suggest that NAFT1 signaling promotes IL‐18 production through the p38 MAPK pathway after tumor inoculation.

### 3.7 NFAT1/IL‐18 Enhances NMDA Receptor Activation in the Spinal Neurons After Tumor Inoculation

**FIGURE 8:**
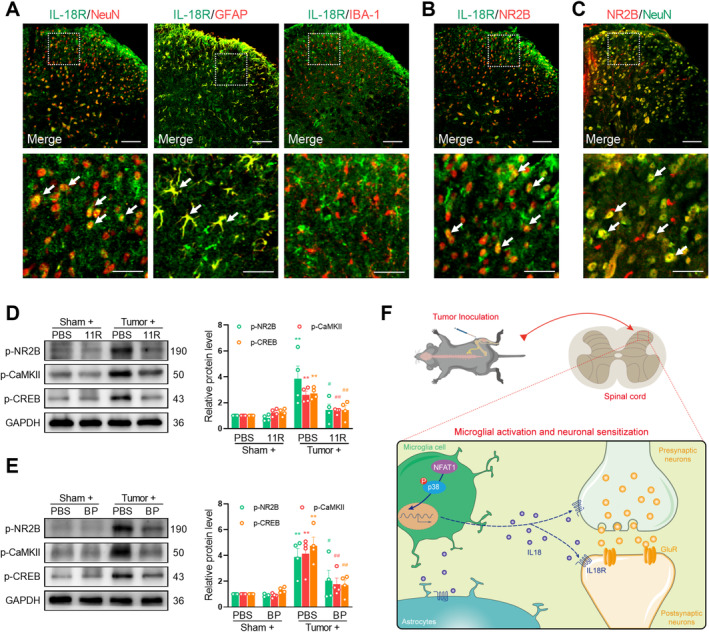
Spinal blockade of the IL‐18/IL‐18R axis suppresses tumor‐induced NMDA receptor activation in the spinal cord. (A) IL‐18R immunoreactivity was primarily colocalized with NeuN and GFAP, but not with IBA‐1. (B, C) NR2B immunoreactivity was colocalized with IL‐18R (B) and NeuN (C) in the spinal dorsal horn. Tissues were collected on day 14 post‐tumor inoculation. Scale bars: 50 μm and 20 μm (zoom). (D, E) Western blot analysis shows that 11R‐VIVIT (D) or IL‐18 BP (E) significantly attenuated tumor‐induced upregulation of p‐NR2B, p‐CaMKII, and p‐CREB protein expression in the spinal dorsal horn. 11R‐VIVIT (1 nM/10 μL, i.t.), IL‐18 BP (1 μg/10 μL, i.t.), or PBS (vehicle control, 10 μL, i.t.) was injected once daily on days 12, 13, and 14 post‐tumor inoculation. Tissues were collected 4 h after the last injection on day 14. Data are expressed as the mean ± SEM. **p < 0.01 versus sham + PBS group; #*p* < 0.05, ##*p* < 0.01 versus tumor + PBS group. *N* = 4 for each group, one‐way ANOVA with post hoc Dunnett's test. (F) Schematic illustration of glia–neuron interactions in the spinal cord dorsal horn in bone cancer pain. NFAT1 is upregulated in spinal microglia in Lewis lung carcinoma‐induced bone cancer pain. Upon activation, microglia synthesize and release IL‐18 through p38 MAPK phosphorylation, leading to enhanced activation of the NMDA receptor and the subsequent Ca^2+^‐dependent signaling in the dorsal horn.

The IL‐18 pathway can facilitate neuronal plasticity through interactions with the neuronal glutamate receptor by binding to its receptor IL‐18R. Consistent with previous studies, the immunoreactivity of IL‐18R primarily colocalized with NeuN and GFAP but not with IBA‐1 (Figure 8A). Immunofluorescence confirmed that IL‐18R colocalized with *N*‐methyl‐D‐aspartate (NMDA) receptor subunit 2B (NR2B) (Figure 8B) in spinal cord neurons (Figure 8C), suggesting that IL‐18 signaling interacts with the NMDA receptor. Extensive expression of NMDA receptors on the postsynaptic neuronal membrane occurs in the spinal cord during the development of chronic pain [33]. After stimulation, NMDA receptors can increase the levels of the intracellular secondary messenger Ca^2+^ and augment the downstream Ca^2+^ signaling molecules, such as CaMKII and CREB, which play crucial roles in the generation and sustenance of central sensitization. Specifically, CaMKII enhances neuronal excitability by phosphorylating AMPA and NMDA receptors, whereas CREB, functioning as a transcription factor, promotes the synthesis of ligands for NMDA receptors [34, 35]. We hypothesize that NFAT1/IL‐18 signaling enables the stimulation of NMDA receptors and Ca^2+^‐triggered signals in spinal neurons. To evaluate the neuronal mechanism of NFAT1/IL‐18 in the development of pain hypersensitivity induced by tumor inoculation, we evaluated the change in the expression of p‐NR2B, p‐CaMKII, and p‐CREB in the spinal dorsal horn by using spinal blockade of NFAT1 or IL‐18. Western blot analysis demonstrated a significant increase in the expression of p‐NR2B, p‐CaMKII, and p‐CREB on day 14 post‐tumor inoculation. However, the intrathecal injection of 11R‐VIVIT (Figure 8D) or IL‐18 BP (Figure 8E) significantly reduced the tumor‐induced expression of p‐NR2B, p‐CaMKII, and p‐CREB. Collectively, these findings suggest that the NFAT1/IL‐18 signaling pathway is involved in tumor‐induced pain hypersensitivity by regulating neuronal activity through modulation of the NMDA receptor (Figure 8F).

## 4 Discussion

In the current study, our results showed persistent upregulation of NFAT1 expression in microglia after LLC inoculation. Blocking NFAT1 decreased neuronal sensitization and microglial activation and relieved mechanical allodynia, thermal hyperalgesia, and spontaneous pain. Moreover, p38 MAPK was confirmed to be an important downstream signal of NFAT1, which was highly expressed in spinal microglia and critical for proinflammatory cytokine IL‐18 production. Additionally, IL‐18 signaling contributed to bone cancer pain by regulating neuronal activity through the NMDA receptor and Ca^2+^‐triggered signals. These results suggest that NFAT1 is a potential target for the treatment of bone cancer pain.

In previous studies, various cancer cells have been injected into the axonal bones of mice or rats to construct animal models that mimic the symptoms and pathology of clinical bone cancer pain [3, 23, 36, 37, 38]. In this study, we inoculated LLC cells into the tibial cavity of mice to induce osteolytic destruction and pathological fractures, accompanied by mechanical allodynia, thermal hyperalgesia, and spontaneous pain, thereby constructing a mouse bone cancer‐induced pain model. Many studies have confirmed microglial activation in different pain models, such as neuropathic and inflammatory pain [32, 39]. However, microglia responses also show diversity in bone cancer‐induced pain models due to differences in sex, animal species, and tumor cell origin [40, 41]. There is no microglial reaction in a female rat bone cancer pain model induced by metastasis tumor cells [42], while microglial activation is detected in male or female rats and mice after inoculation of specific carcinoma cells, including Walker 256 mammary carcinoma cells and Lewis lung carcinoma cells [3, 23, 43, 44]. In our study, we found that inoculation with LLC cells resulted in a significant activation of microglia in male mice.

NFAT has been discovered in nuclear extracts from activated T cells, later confirmed to be expressed in various cells [8, 45, 46, 47, 48]. NFAT isoforms, such as NFAT3 and NFAT1, play a crucial role in neuronal survival and apoptosis [49, 50]. Recent studies have proven that NFAT1 may be involved in proinflammatory responses and microglial proliferation [9, 11, 51, 52]. We found that NFAT1 expression increased rapidly and continuously in spinal microglia after tumor inoculation in male mice. Our data further confirmed that continuous intrathecal injections of NFAT1 inhibitors suppressed tumor‐mediated bone cancer pain, indicating that NFAT1 plays an important role in the development and progression of bone cancer pain in male mice. However, the role of NFAT1 in bone cancer pain in female mice is worth being determined in future studies. Notably, the NFAT pathway is pivotal in various functions of the peripheral nervous system [47, 53], and we cannot rule out the contribution of peripheral NFAT1 in bone cancer pain. In addition, our current study cannot exclude the possibility that intrathecal injection of an inhibitor may also block NFAT1 pathway activity in dorsal root ganglion neurons, thereby partially contributing to the alleviation of bone cancer pain.

Research has indicated that the MAPK signaling pathway is significant in neuronal plasticity and the transmission of nociceptive signals by modulating gene expression, protein production, and receptor levels [54]. P38 MAPK is selectively activated in spinal microglia after spinal nerve injury or neonatal colonic inflammation, and pharmacological inhibition of p38 MAPK can attenuate both neuropathic pain and inflammatory pain [55, 56, 57]. The expression of p38 MAPK has significantly been confirmed to diminish in Nfat1^−/−^ mice after spinal nerve ligation [11]. Our results further confirmed that p38 MAPK was exclusively expressed in the spinal microglia and increased after tumor injection. We then found that the p38 MAPK inhibitor SB239063 markedly alleviated bone cancer pain. Moreover, the tumor‐induced increase in p38 MAPK expression was significantly inhibited by the application of the NFAT1 inhibitor 11R‐VIVIT. Thus, spinal microglial p38 MAPK is a key downstream of the NFAT1 signaling pathway in bone cancer pain.

Consistent with previous reports, IL‐18 was exclusively expressed in the spinal microglia after tumor injection [13, 16, 43, 58]. Blocking NFAT1 or p38 MAPK could markedly suppress the expression of IL‐18 after tumor injection. Thus, NAFT1 signaling could promote IL‐18 production through the p38 MAPK pathway in the spinal microglia after tumor inoculation. However, NFATc interacts with its transcriptional partners, such as activator protein‐1 in the nucleus, to directly regulate microglial gene expression in vitro and in vivo, including cytokines and cell surface receptors [11, 51]. As a transcription factor, we cannot rule out that NFAT1 may directly upregulate IL‐18 expression. NFAT1 activity is regulated by various post‐transcriptional modifications, including acetylation [59], SUMOylation [60], and demethylation [11], which can significantly affect the stability and transcriptional activity of NFAT1. Research has confirmed that demethylation of the NFAT1 promoter increases CD11b expression, which promotes microglial activation and the expression of proinflammatory cytokine [11]. However, whether NFAT is regulated by post‐transcriptional modifications following tumor inoculation remains to be further investigated.

IL‐18 interacts with its heterodimeric receptor (IL‐18R), triggering a cascade reaction, thereby participating in the incidence of chronic pain [25, 43, 61, 62, 63]. The IL‐18–IL‐18R axis could regulate the phosphorylation of NMDA receptors in the neurons through Src kinase [25]. Exogenous IL‐18 could induce pain behavior after intrathecal injection, and increase the frequency of mEPSCs in spinal cord neurons [43]. Consistently, our data demonstrated that IL‐18 was exclusively expressed in the spinal microglia, whereas IL‐18R was distributed in the spinal neuron and colocalized primarily with NR2B. Repeated intrathecal injection of IL‐18 BP effectively attenuated the tumor‐induced activation of NR2B and the subsequent Ca^2+^‐dependent signals as well as tumor‐induced bone cancer pain, suggesting that NFAT1/IL‐18 could regulate synaptic plasticity by interacting with NMDA receptors.

IL‐18R was expressed in neurons [25, 43], we also found that IL‐18R was expressed in astrocytes in the spinal dorsal horn following tumor injection. A similar observation has been reported in rats with fibromyalgia [64]. Moreover, increased CX3CL1 promotes the synthesis of IL‐18 in microglia in the sciatic nerve chronic constrictive injury model, which stimulates IL‐18R on adjacent astrocytes through a paracrine pathway and regulates downstream molecules through the NF‐κB signaling pathway [65]. Therefore, the IL‐18R pathway might be partially involved in regulating astrocyte signaling in bone cancer pain.

## 5 Conclusion

This study provides a new strategy for the clinical management of bone cancer pain. NFAT1/IL‐18 pathway‐mediated microglia–neuron interactions may facilitate and improve processing of bone cancer pain. Consequently, the inhibition of the NFAT1 signaling pathway may be effective in the prevention and/or alleviation of bone cancer pain.

## Author Contributions

X.C., and Z.P. conceived the project and supervised all experiments. Y.Z., Z.W., J.Z., F.L., M.Z., J.Z., Q.C., and D.Z. performed the experiments. X.C., Y.Z., Y.C., J.N., and Z.X. analyzed data. F.L., G.S., and Z.P. wrote the manuscript. All authors reviewed and approved the final manuscript.

## Conflicts of Interest

The authors declare no conflicts of interest.

## Supporting information


Data S1.

## Data Availability

The data that support the findings of this study are available from the corresponding author upon reasonable request.
