# Supplementary material for: NFAT1 Signaling Contributes to Bone Cancer Pain by Regulating IL‐18 Expression in Spinal Microglia
Source: CNS Neurosci Ther. 2025 Feb 17;31(2):e70222. doi: 10.1111/cns.70222 (PMC11831200; doi:10.1111/cns.70222)
Supplement: Supplementary file 1 — Data S1. [file CNS-31-e70222-s001.zip › 3.Supplementary materials 01.pdf]

Figure1. G

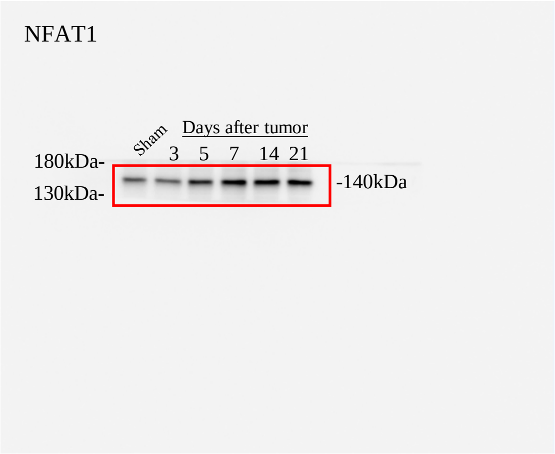
NFAT1
Sham
Days after tumor
3 5 7 14 21
180kDa-
130kDa-
-140kDa

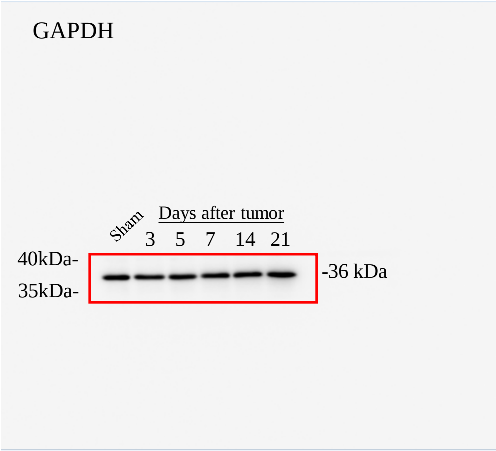
GAPDH
Sham
Days after tumor
3 5 7 14 21
40kDa-
35kDa-
-36 kDa

Figure4. A

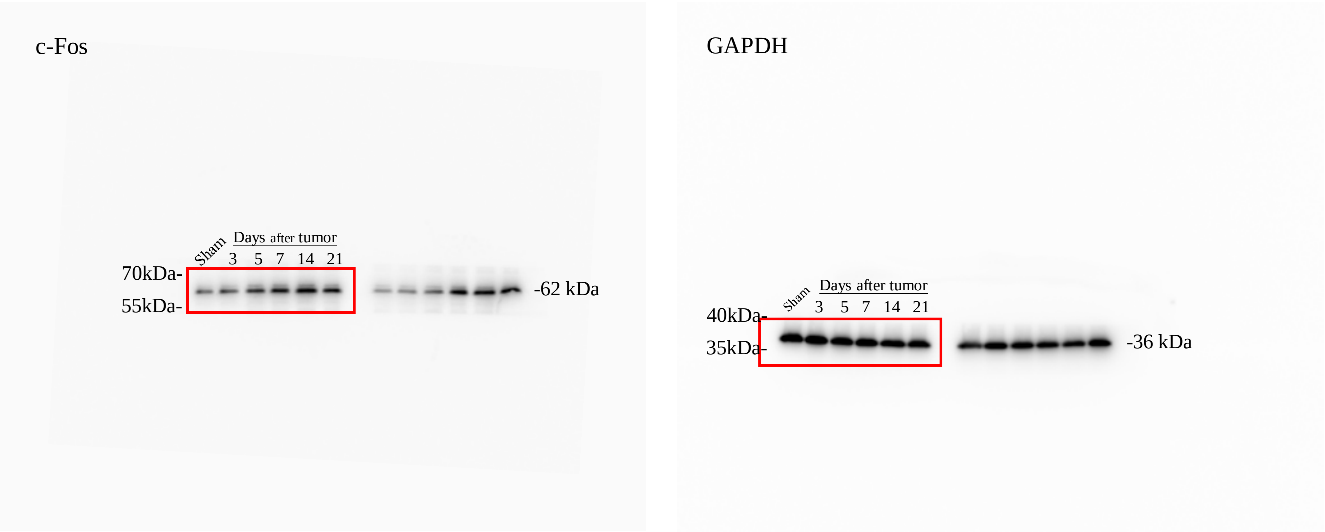
c-Fos
Sham
Days after tumor
3 5 7 14 21
70kDa-
55kDa-
-62 kDa
GAPDH
Sham
Days after tumor
3 5 7 14 21
40kDa-
35kDa-
-36 kDa

Figure4. B

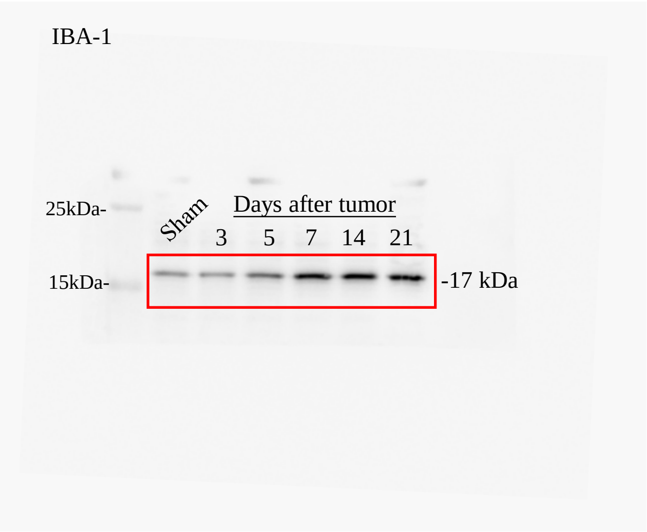
IBA-1
25kDa-
15kDa-
Sham
Days after tumor
3
5
7
14
21
-17 kDa

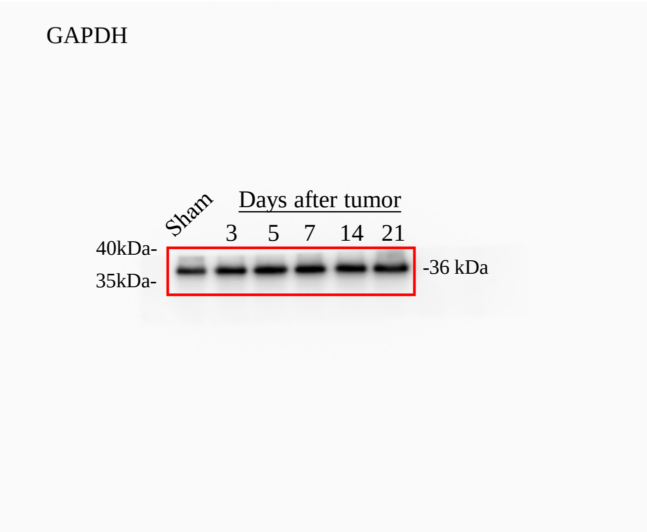
GAPDH
40kDa-
35kDa-
Sham
Days after tumor
3
5
7
14
21
-36 kDa

Figure4. C

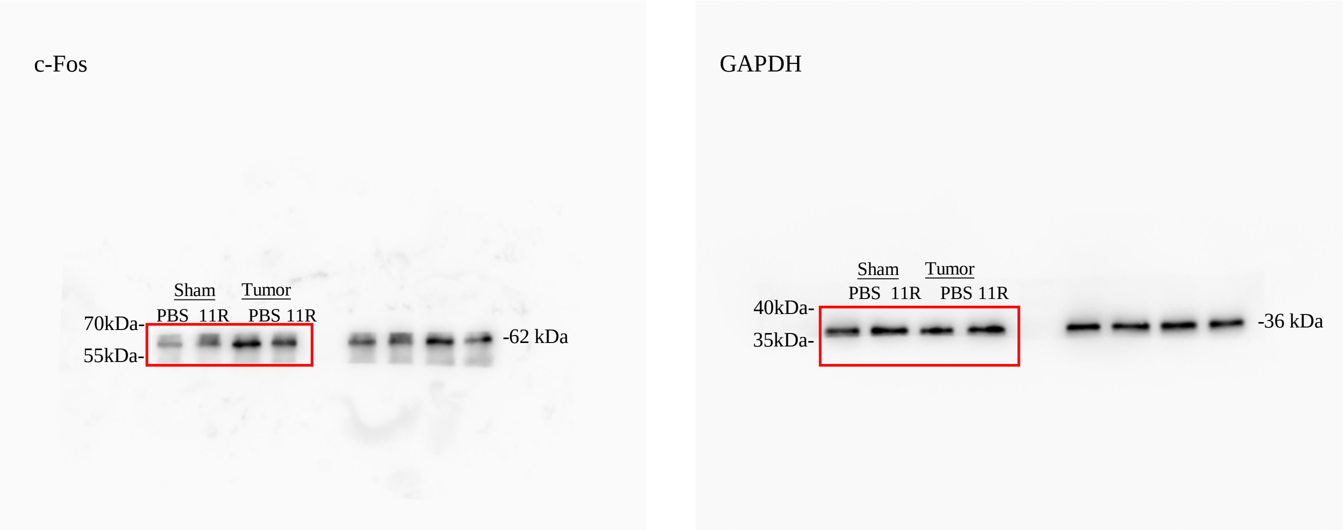
c-Fos
Sham
Tumor
PBS 11R PBS 11R
70kDa-
55kDa-
-62 kDa
GAPDH
Sham
Tumor
PBS 11R PBS 11R
40kDa-
35kDa-
-36 kDa

Figure4. D

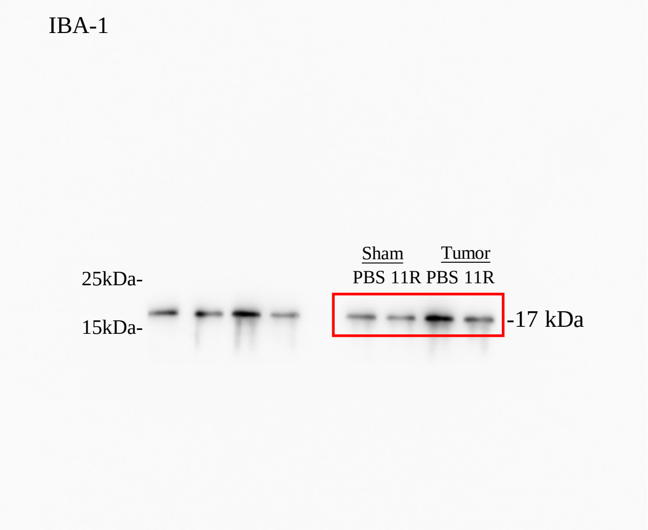
IBA-1
Sham
Tumor
PBS 11R PBS 11R
25kDa-
15kDa-
-17 kDa

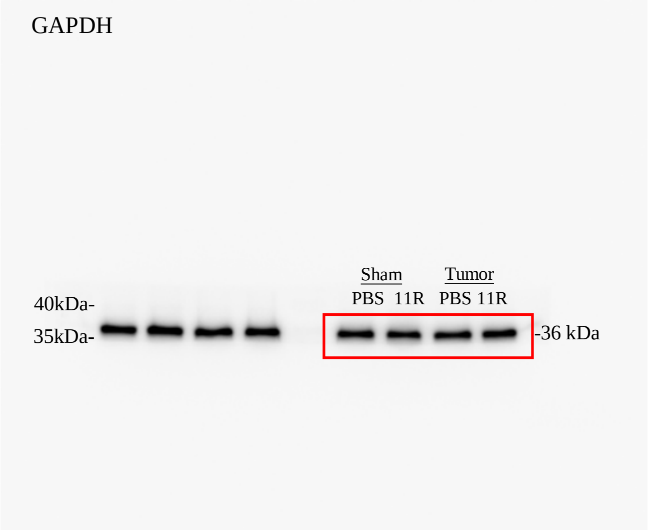
GAPDH
Sham
Tumor
PBS 11R PBS 11R
40kDa-
35kDa-
-36 kDa

Figure5. A

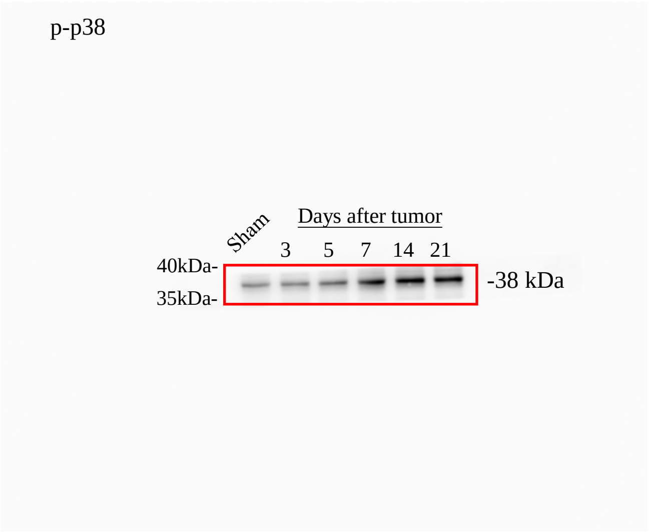

p-p38
Sham
Days after tumor
3 5 7 14 21
40kDa-
35kDa-
-38 kDa

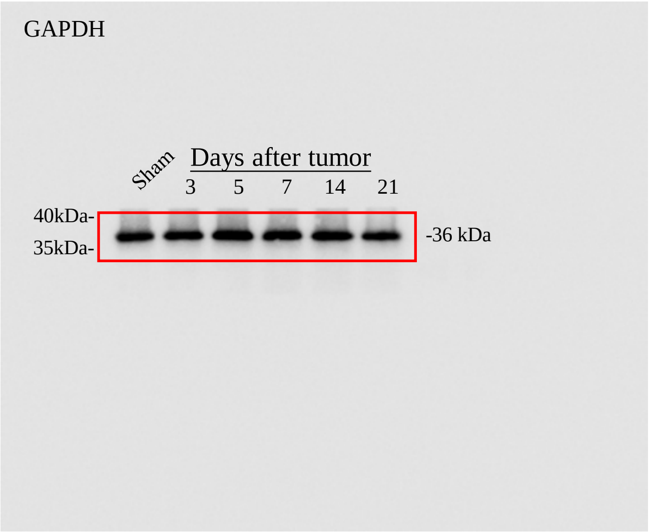

GAPDH
Sham
Days after tumor
3 5 7 14 21
40kDa-
35kDa-
-36 kDa

Figure5. C

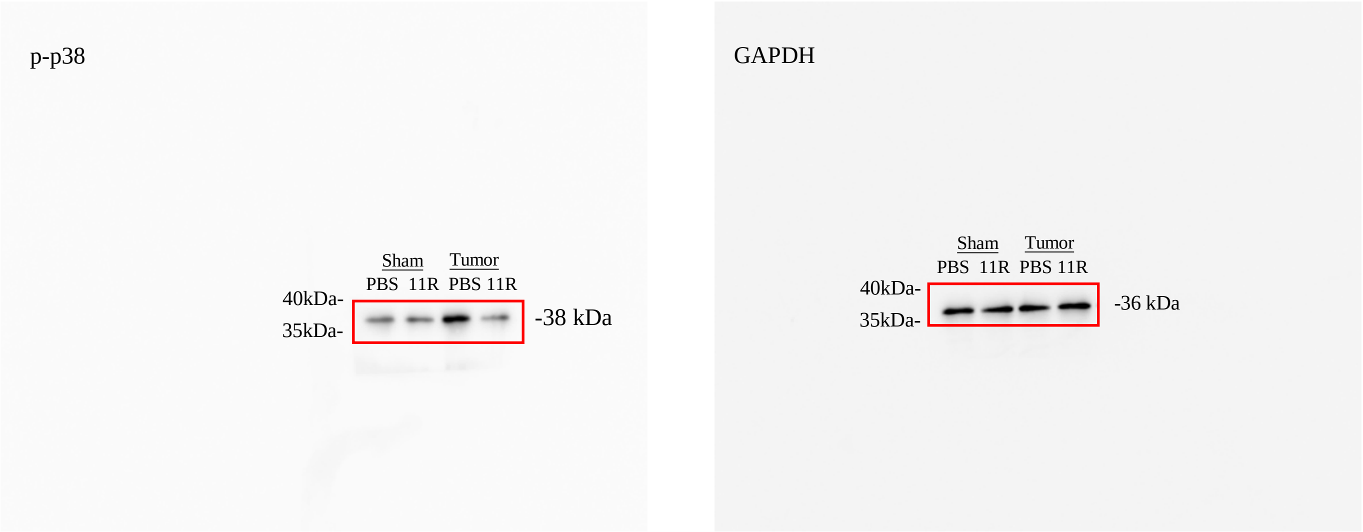
p-p38
Sham
Tumor
PBS 11R PBS 11R
40kDa-
35kDa-
-38 kDa
GAPDH
Sham
Tumor
PBS 11R PBS 11R
40kDa-
35kDa-
-36 kDa

Figure7. A

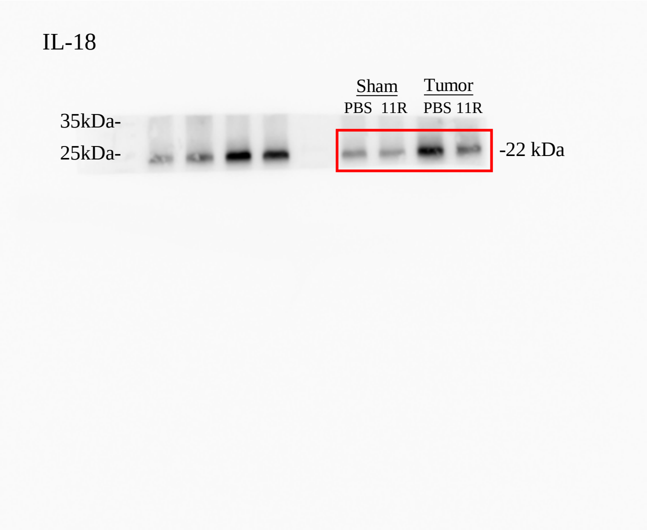
IL-18
Sham
Tumor
PBS 11R
PBS 11R
35kDa-
25kDa-
-22 kDa

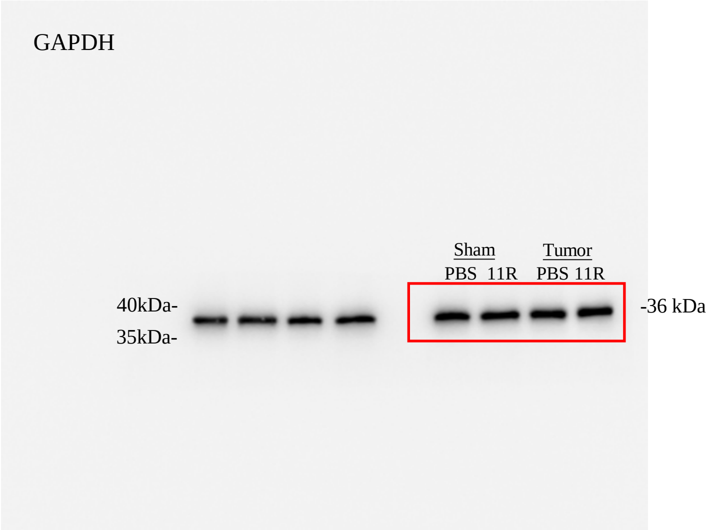
GAPDH
Sham
Tumor
PBS 11R
PBS 11R
40kDa-
35kDa-
-36 kDa

Figure7. B

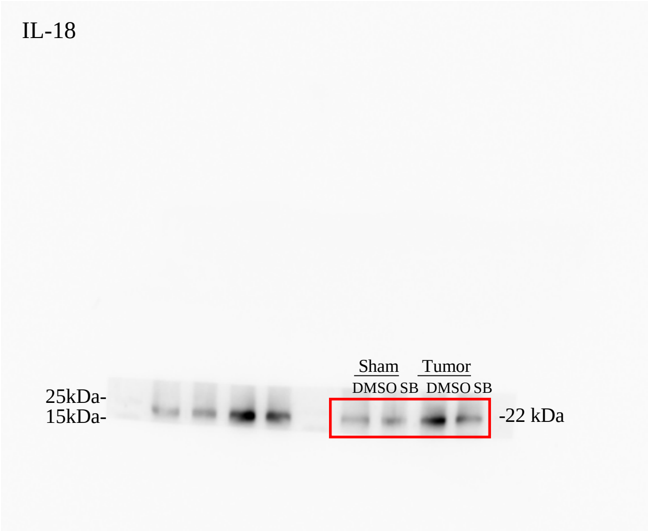
IL-18
Sham
Tumor
DMSO SB DMSO SB
25kDa-
15kDa-
-22 kDa

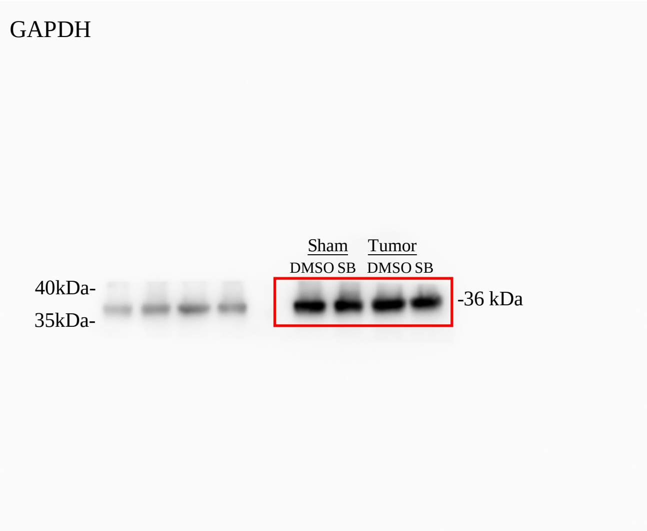
GAPDH
Sham
Tumor
DMSO SB DMSO SB
40kDa-
35kDa-
-36 kDa

Figure8. D

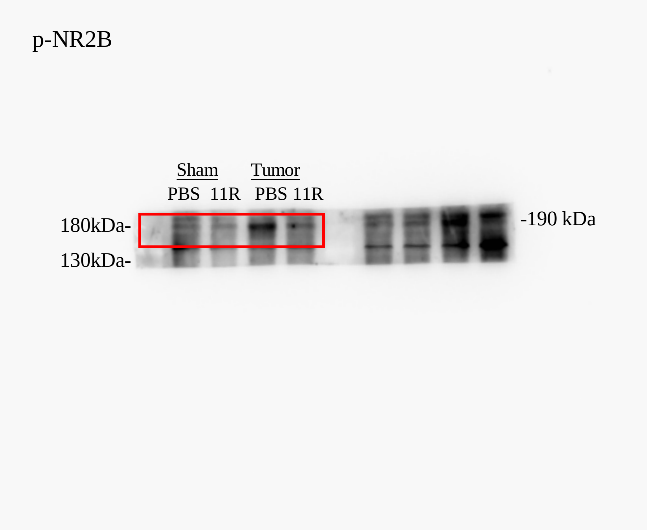

p-NR2B
Sham
Tumor
PBS 11R PBS 11R
180kDa-
130kDa-
-190 kDa

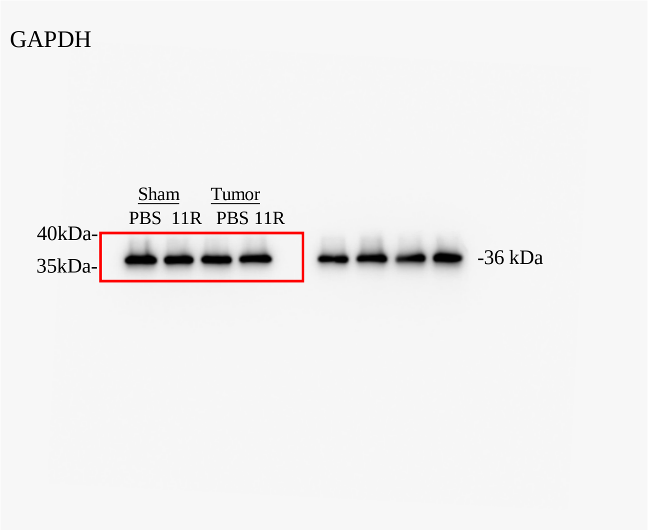

GAPDH
Sham
Tumor
PBS 11R PBS 11R
40kDa-
35kDa-
-36 kDa

Figure8. D

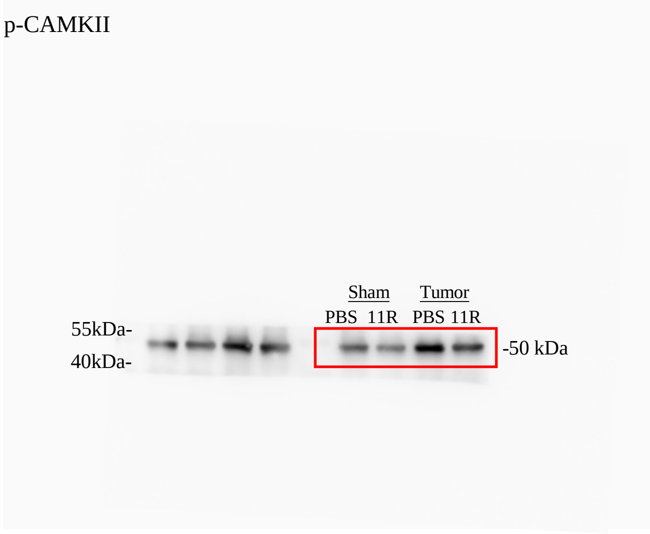
p-CAMKII
Sham
Tumor
PBS 11R
PBS 11R
55kDa-
40kDa-
-50 kDa

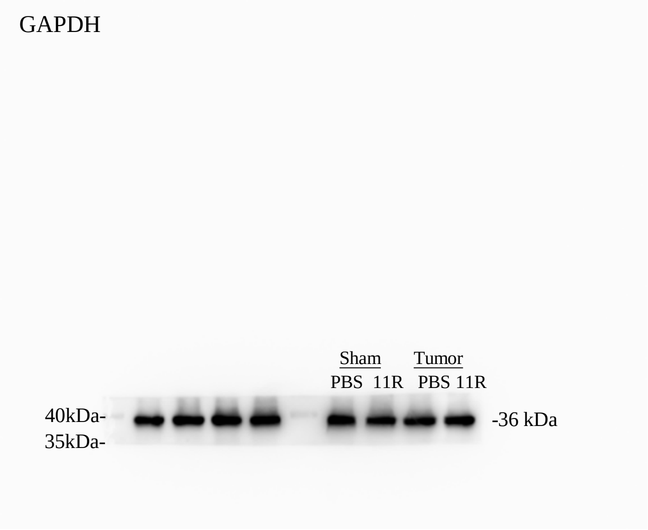
GAPDH
Sham
Tumor
PBS 11R
PBS 11R
40kDa-
35kDa-
-36 kDa

Figure8. D

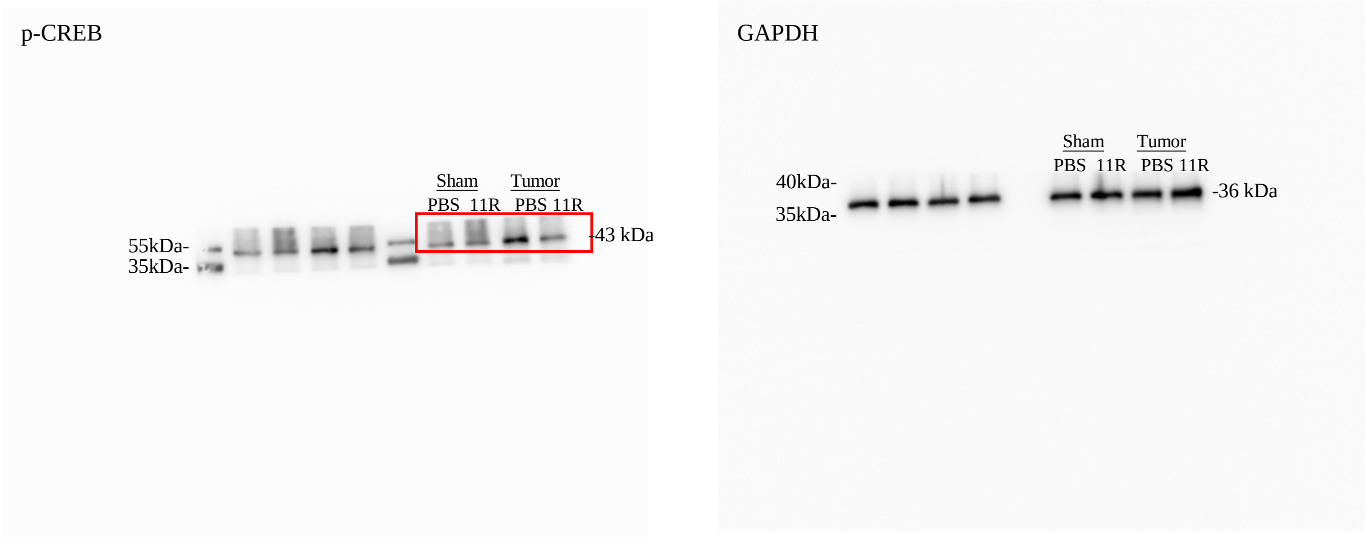
p-CREB
Sham
Tumor
PBS 11R PBS 11R
55kDa-
35kDa-
-43 kDa
GAPDH
Sham
Tumor
PBS 11R PBS 11R
40kDa-
35kDa-
-36 kDa

Figure8. E

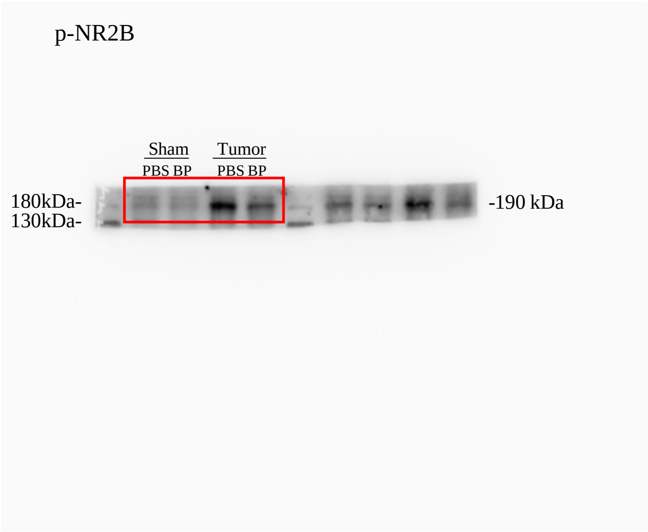
p-NR2B
Sham
Tumor
PBS BP
PBS BP
180kDa-
130kDa-
-190 kDa

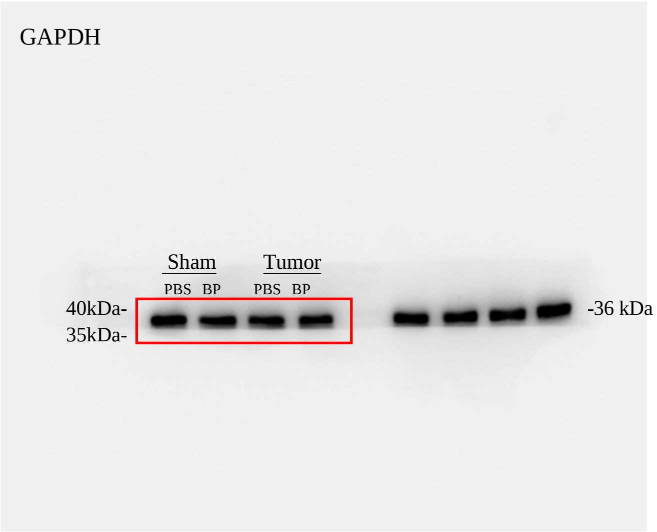
GAPDH
Sham
Tumor
PBS BP
PBS BP
40kDa-
35kDa-
-36 kDa

Figure8. E

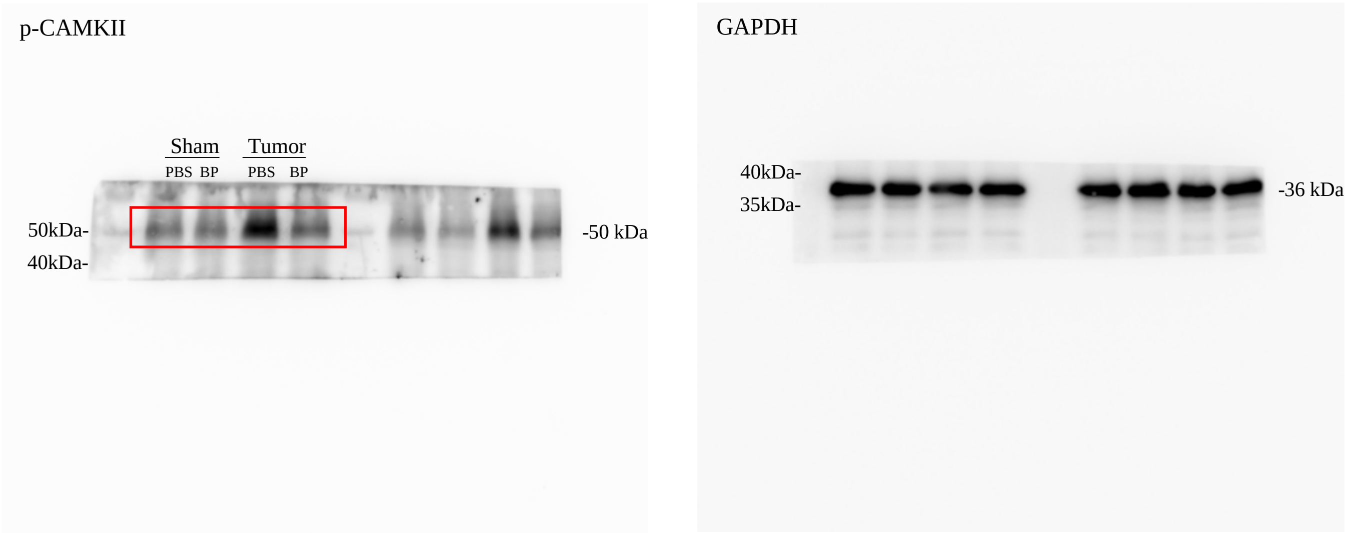
p-CAMKII
Sham
Tumor
PBS BP
PBS BP
50kDa-
40kDa-
-50 kDa
GAPDH
40kDa-
35kDa-
-36 kDa

Figure8. E

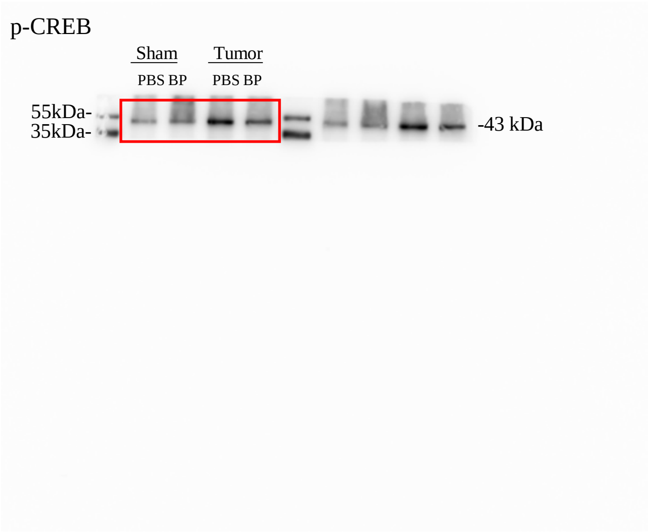
p-CREB
Sham
Tumor
PBS BP
PBS BP
55kDa-
35kDa-
-43 kDa

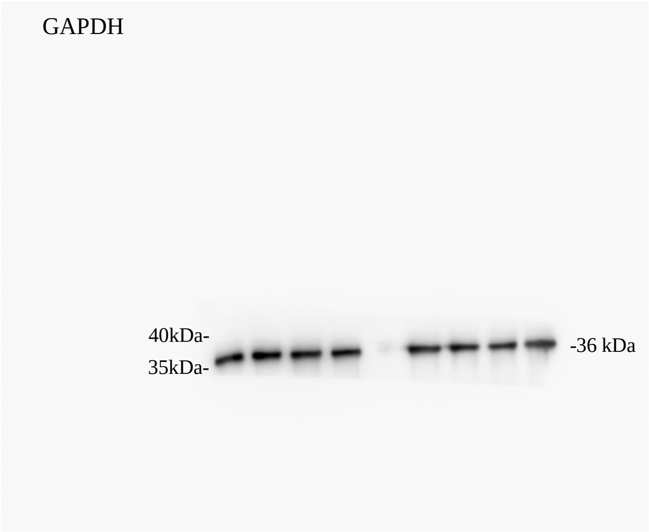
GAPDH
40kDa-
35kDa-
-36 kDa
