# Supplementary material for: NFAT1 Signaling Contributes to Bone Cancer Pain by Regulating IL‐18 Expression in Spinal Microglia
Source: CNS Neurosci Ther. 2025 Feb 17;31(2):e70222. doi: 10.1111/cns.70222 (PMC11831200; doi:10.1111/cns.70222)
Supplement: Supplementary file 1 — Data S1. [file CNS-31-e70222-s001.zip › cns70222-sup-0001-TableS1.docx]

| Figure | Panel | Interaction | | | Time | | | Treatment | | |
| --- | --- | --- | --- | --- | --- | --- | --- | --- | --- | --- |
|  |  | DFn,DFd | F | P | DFn,DFd | F | P | DFn,DFd | F | P |
| 1 | C | 10, 70 | 8.964 | P<0.0001 | 5, 35 | 3.684 | P=0.0088 | 2, 14 | 49.95 | P<0.0001 |
| 1 | D | 10, 70 | 6.49 | P<0.0001 | 5, 35 | 10.75 | P<0.0001 | 2, 14 | 48.83 | P<0.0001 |
| 3 | A | 24, 168 | 5.135 | P<0.0001 | 8, 56 | 14.27 | P<0.0001 | 3, 21 | 30.89 | P<0.0001 |
| 3 | B | 24, 168 | 5.934 | P<0.0001 | 8, 56 | 12.94 | P<0.0001 | 3, 21 | 69.49 | P<0.0001 |
| 3 | C | 24, 168 | 2.549 | P=0.0054 | 8, 56 | 5.963 | P<0.0001 | 3, 21 | 31.37 | P<0.0001 |
| 3 | D | 24, 168 | 3.649 | P<0.0001 | 8, 56 | 9.548 | P<0.0001 | 3, 21 | 33.67 | P<0.0001 |
| 5 | F | 24, 168 | 2.456 | P=0.0005 | 8, 56 | 6.054 | P<0.0001 | 3, 21 | 26.57 | P<0.0001 |
| 5 | G | 24, 168 | 3.149 | P<0.0001 | 8, 56 | 7.647 | P<0.0001 | 3, 21 | 68.51 | P<0.0001 |
| 7 | F | 24, 168 | 2.484 | P=0.0004 | 8, 56 | 7.075 | P<0.0001 | 3, 21 | 35 | P<0.0001 |
| 7 | G | 24, 168 | 2.674 | P=0.0001 | 8, 56 | 8.779 | P<0.0001 | 3, 21 | 35.27 | P<0.0001 |

Table S1. Two-way RM ANOVA

| Figure | Panel | Factor | Treatment | | |
| --- | --- | --- | --- | --- | --- |
|  |  |  | DFn,DFd | F | P |
| 1 | E | Behavior | 2, 21 | 60.86 | P<0.0001 |
| 1 | F | NFAT1 | 5, 18 | 22 | P<0.0001 |
| 1 | G | NFAT1 | 5, 18 | 6.498 | P=0.0013 |
| 3 | E | Behavior | 3, 28 | 65.99 | P<0.0001 |
| 3 | F | Behavior | 3, 28 | 41.24 | P<0.0001 |
| 4 | A | c-Fos | 5, 18 | 11.37 | P<0.0001 |
| 4 | B | IBA-1 | 5, 18 | 6.421 | P=0.0014 |
| 4 | C | c-Fos | 3, 12 | 16.26 | P=0.0002 |
| 4 | D | IBA-1 | 3, 12 | 11.54 | P=0.0008 |
| 4 | F | c-Fos | 2, 21 | 28.39 | P<0.0001 |
| 4 | H | IBA-1 | 2, 21 | 43.33 | P<0.0001 |
| 5 | A | p-p38 | 5, 18 | 9.988 | P=0.0001 |
| 5 | C | p-p38 | 3, 12 | 11.44 | P=0.0008 |
| 5 | E | p-p38 | 2, 21 | 77.32 | P<0.0001 |
| 5 | H | Behavior | 2, 21 | 41.97 | P<0.0001 |
| 7 | A | IL-18 | 3, 12 | 25.94 | P<0.0001 |
| 7 | B | IL-18 | 3, 12 | 15.01 | P=0.0002 |
| 7 | H | Behavior | 2, 21 | 47.61 | P<0.0001 |
| 8 | D | p-NR2B | 3, 12 | 6.649 | P=0.0068 |
| 8 | D | p-CaMKII | 3, 12 | 18.64 | P<0.0001 |
| 8 | D | p-CREB | 3, 12 | 12.5 | P=0.0005 |
| 8 | E | p-NR2B | 3, 12 | 9.115 | P=0.0020 |
| 8 | E | p-CaMKII | 3, 12 | 13.23 | P=0.0004 |
| 8 | E | p-CREB | 3, 12 | 19.56 | P<0.0001 |

Table S2. Ono-way ANOVA
